# Global burden and projections of cardiometabolic diseases attributable to high alcohol use: a comparative risk assessment based on the GBD 2021 study

**DOI:** 10.3389/fnut.2026.1698730

**Published:** 2026-03-06

**Authors:** Yuqing Tang, Derong Lin, Honglin Xu, Liman Xu, Sien Guo, Xuankun Zheng, Meiyi Su, Kefeng Zeng, Wenwei Feng, Jianfeng Ye, Lei Wang

**Affiliations:** 1 Dongguan Hospital of Traditional Chinese Medicine, Guangzhou University of Chinese Medicine, Dongguan, China; 2 The Second Clinical College, Guangzhou University of Chinese Medicine, Guangzhou, China; 3 Department of Cardiology, Dongguan Hospital of Traditional Chinese Medicine, Dongguan, China; 4 Guangdong Provincial Hospital of Chinese Medicine, Guangzhou, China

**Keywords:** age–period–cohort model, cardiometabolic diseases, global burden of disease, high alcohol use, projections

## Abstract

**Background and aims:**

Cardiometabolic diseases (CMDs), including cardiovascular disease (CVD) and type 2 diabetes mellitus (T2DM), remain major global health challenges. High alcohol use (HAU) is a modifiable risk factor. This study quantified the global, regional, and temporal trends in the burdens of CMDs attributable to HAU from 1990 to 2021 and projected trends to 2040.

**Methods and results:**

Global Burden of Disease 2021 (GBD 2021) estimates for 204 countries and territories were analyzed to quantify HAU-attributable deaths, disability-adjusted life years (DALYs), and age-standardized mortality and DALY rates (ASMRs, ASDRs). HAU-attributable burdens were interpreted as model-based scenario estimates under the GBD 2021 comparative risk assessment framework, and not as individual-level causal effects. Associations with the Sociodemographic Index (SDI) were assessed. Trends in rates were summarized using estimated annual percentage change (EAPC) as a descriptive metric derived from log-linear regression on GBD age-standardized rate estimates, and projections were generated with a Bayesian age–period–cohort model. Although global HAU exposure declined, HAU-attributable deaths and DALYs from CVD and T2DM increased, with higher burdens among males and middle-aged adults. From 1990 to 2021, EAPCs based on age-standardized rates suggested modest declines in HAU-attributable CVD-related ASMR and ASDR (−1.53 and −1.31), whereas HAU-attributable T2DM ASMR and ASDR showed an overall increasing tendency (0.48 and 1.83), particularly in low- and middle-SDI regions. Eastern and Central Europe had the highest HAU-attributable CVD burden; Oceania and Central Latin America had the highest T2DM burden. By 2040, under a business-as-usual continuation of recent trends, scenario-based projections suggest that deaths attributable to HAU could rise substantially (on the order of 70% for CVD and nearly three-fold for T2DM), with widening sex disparities and greater quantitative uncertainty for CVD than for T2DM.

**Conclusion:**

Despite declining alcohol exposure, the burden of CMDs attributable to HAU is escalating–especially for T2DM, males, and populations in low and middle SDI regions. Region-specific interventions and stronger alcohol-control policies are urgently needed.

## 1 Introduction

Cardiometabolic diseases (CMDs), comprising primarily type 2 diabetes mellitus (T2DM) and cardiovascular disease (CVD), represent a group of chronic conditions characterized by metabolic dysregulation and cardiovascular impairment (1, 2). Population aging, increasingly sedentary lifestyles, and the widespread adoption of energy-dense diets have together driven sustained rises in both the incidence and mortality of CMDs, putting substantial pressure on health care systems worldwide (3). Estimates from the Global Burden of Disease (GBD) study indicate that the burden of T2DM and CVD has continued to grow over recent decades, with the sharpest increases observed in low and middle income countries and in rapidly urbanizing regions (4, 5).

High alcohol use (HAU) is a major modifiable risk factor linked to a broad spectrum of metabolic and cardiovascular derangements (6). Epidemiological evidence shows that chronic excessive alcohol consumption promotes T2DM and CVD through pathways involving hepatic metabolic dysfunction, insulin resistance, dyslipidaemia, and alcohol-induced structural and functional myocardial changes (7, 8). HAU frequently co-occurs with other deleterious behaviors such as hyper-caloric diets, physical inactivity, and tobacco use, and this combination increases cardiometabolic risk (9). Within the GBD framework, HAU is classified as a key behavioral risk factor and is operationalized as alcohol intake that exceeds the theoretical minimum risk exposure level (TMREL) per day (10). Despite targeted control policies in some jurisdictions, the global prevalence of HAU remains persistently high (11).

Although numerous studies have examined the cardiovascular consequences of alcohol consumption (12), there remains a lack of integrated appraisals of the combined burden of CMDs attributable to HAU, particularly analyses that jointly consider CVD and T2DM and integrate regional disparities and future projections within a single analytical framework. Previous GBD alcohol publications have provided foundational evidence by quantifying alcohol-attributable burden across broad cause groupings (13) and by characterizing population-level risks by drinking amount across geography, age, sex and year (14). However, these analyses were not designed to deliver a dedicated cardiometabolic synthesis that directly juxtaposes HAU-attributable CVD and T2DM within a single, harmonized framework. Building on the GBD 2021 comparative risk assessment as a secondary analysis of modeled data, this study aims to estimate the global, regional and temporal burden of CMDs attributable to HAU during 1990–2021. All estimates are stratified by sex, age group and Sociodemographic Index (SDI) quintile, and a Bayesian age–period–cohort (BAPC) model is applied to generate business-as-usual projections through 2040. Within this framework, the present secondary analysis adds three elements beyond prior GBD alcohol work: (i) explicit cardiometabolic integration that harmonizes and directly compares HAU-attributable total CVD versus T2DM within a single framework; (ii) systematic characterization of SDI- and region-specific heterogeneity stratified by sex and age; and (iii) business-as-usual, scenario-based projections to 2040 using a BAPC model, alongside transparent reporting of uncertainty and reproducible query specifications and code. The findings are intended to inform public-health priority-setting and to support the design of context-sensitive alcohol-related prevention strategies worldwide.

## 2 Materials and methods

### 2.1 Study data

All data were obtained from the Global Burden of Disease 2021 (GBD 2021) database, which is curated by the Institute for Health Metrics and Evaluation (IHME) at the University of Washington. GBD 2021 combines systematic reviews, statistical modeling and multiple data streams to generate harmonized estimates for 371 diseases and 88 risk factors across 204 countries and territories. Core epidemiological indicators–including mortality, prevalence, disability-adjusted life-years (DALYs) and risk-attributable burden–were extracted using the GBD Results Tool^1^ and the GBD Compare platform. GBD 2021 estimates for 204 countries and territories were analyzed to quantify HAU-attributable deaths, DALYs and age-standardized rates (ASMRs, ASDRs). All HAU-attributable deaths, DALYs and age-standardized rates were taken directly from the GBD 2021 comparative risk assessment for the risk factor “alcohol use” and its linked CVD and T2DM cause–risk pairs. Further details on exposure definitions, relative risk functions, PAF formulation, and data extraction are provided in the Supplementary Method 2. The full GBD 2021 query specifications, extracted aggregate datasets, and R scripts used to compute EAPCs and fit the BAPC models are provided in the Supplementary materials.

This study used only publicly available, de-identified aggregate data from GBD 2021 and was confirmed by the Ethics Committee of Dongguan Hospital of Traditional Chinese Medicine to meet the criteria for exemption from institutional ethical review and informed consent requirements.

### 2.2 Study population and disease definitions

CMDs were defined as CVD and T2DM. In this study, cardiometabolic burden was assessed for T2DM and total CVD, following the GBD cause hierarchy. Consistent with the GBD 2021 comparative risk assessment for alcohol use, all analyses were restricted to populations aged ≥ 15 years, using 5-years age groups from 15 to 19 years to ≥95 years. Total CVD includes several subtypes, such as ischemic heart disease, stroke subtypes, and other cardiovascular conditions. These subtypes may have different relationships with alcohol exposure, but they were not analyzed separately in this study. Within the GBD nosology, CVD is a Level 2 category, whereas T2DM is Level 3. Disease definitions and estimation frameworks used in the GBD collaboration have been described and validated in detail previously.

In GBD 2021, CVD comprises ischemic heart disease, heart failure, stroke, atrial fibrillation and flutter, hypertensive heart disease, and related conditions. Coding followed the International Classification of Diseases, 10th Revision (ICD-10), and cause-of-death attribution adhered to World Health Organization criteria. The aggregate cardiovascular burden attributable to HAU was calculated by summing estimates across all CVD subtypes.

Type 2 diabetes mellitus was defined as a fasting plasma glucose concentration ≥126 mg/dL (7.0 mmol/L) or a self–reported history of diabetes treatment. Epidemiological inputs were drawn from population-based surveys, systematic literature reviews, and cohort studies. Non-fatal outcomes were modeled with DisMod-MR 2.1, a Bayesian meta-regression tool that synthesizes heterogeneous data while propagating uncertainty.

### 2.3 Definition of high alcohol use

High alcohol use was defined according to the GBD comparative risk assessment framework as alcohol consumption in excess of the TMREL (10, 14). In the GBD 2021 CRA outputs analyzed here, TMREL is the GBD-defined, stratum-specific counterfactual (by age/sex/location/year) rather than a universally fixed 0 g/day level. In parallel, IARC classifies alcohol as a Group 1 carcinogen, and WHO public health guidance highlights that no level of alcohol consumption is risk-free, particularly for several cancers; however, this public-health framing should not be conflated with the TMREL specification used in the GBD risk assessment. This threshold comes from meta-analyses that delineate continuous dose–response relationships between alcohol exposure and disease-specific relative risks. As it reflects long-term mean intake rather than drinking patterns, this exposure metric does not distinguish episodic heavy drinking from regular moderate use and may introduce directional bias in binge-prevalent settings.

Exposure distributions were constructed from self-reported drinking data from population-based surveys, supplemented by information from epidemiological investigations, the World Health Organization Global Health Observatory, national nutrition surveys, and official statistics archived in the Global Health Data Exchange (GHDx). Reported quantities were standardized to grams of pure ethanol using established conversion factors.

The prevalence of HAU by age, sex, country, and year was estimated using a spatiotemporal Gaussian process regression (ST-GPR) model. This Bayesian framework uses spatial autocorrelation and temporal trends and allows for data sparsity, and it provides consistent exposure estimates with quantified uncertainty.

### 2.4 Statistical analysis

The burden of CVD and T2DM attributable to HAU was summarized using counts (deaths and DALYs), crude rates per 100,000 population, their 95 % uncertainty intervals (UIs), and the corresponding ASMRs and ASDRs. Uncertainty intervals reflect joint variation propagated from exposure estimation, relative-risk inputs, and attribution steps within the GBD framework. Within the GBD comparative risk assessment framework, population-attributable burdens were estimated by combining stratified exposure distributions with multivariable-adjusted relative risks synthesized in hierarchical models, and by contrasting the observed burden with the counterfactual burden expected if exposure were set to the TMREL, as specified in the GBD framework and varying by age and location, rather than being treated as a universal fixed value. Accordingly, “HAU-attributable” burden represents the excess burden attributable to exposure above the GBD TMREL counterfactual, and should not be interpreted as the burden under abstinence or a universal 0 g/day counterfactual. These quantities are model-based scenario estimates under stated assumptions and do not constitute individual-level causal effects. Temporal trends in disease burden were assessed using the estimated annual percentage change (EAPC), derived from log-linear regression models of ASR over time [ln(ASR) = α + β × year + ε]. The EAPC and its 95% confidence interval (CI) were calculated as: EAPC = 100 × [exp(β) − 1]. A positive EAPC with a 95% CI entirely above zero indicated a significant upward trend, a negative EAPC with a 95% CI entirely below zero indicated a downward trend, and a 95% CI spanning zero indicated no significant change. Because these models were fitted to point estimates of ASR and assumed approximate log-linear behavior over time, we used EAPCs as simple summaries of long-term trends rather than precise annualized changes. Small positive or negative values near zero were treated as approximate stability, and we did not over-emphasize them as strong evidence of increase or decrease. The 95% CIs around EAPCs therefore capture only regression uncertainty conditional on the GBD central estimates and do not propagate the full posterior uncertainty in the underlying GBD rates.

The association between ASDR and the SDI was examined using Spearman’s rank correlation, with statistical significance defined as a two-sided *P* < 0.05. SDI is a composite country-level index that combines information on income, education and fertility. In this study, correlations with SDI are ecological and describe patterns between countries only. They should not be interpreted as evidence of individual-level causal relationships or of specific mechanisms. Stratified analyses were conducted according to the GBD framework: five SDI quintiles (high, high-middle, middle, low-middle, low), 21 geographic regions, and 204 countries and territories. Age was grouped into 5-years intervals from 15 to 19 years to ≥95 years. Forecasts of HAU-attributable CVD and T2DM mortality for 2022–2040 were generated using a BAPC model with a Poisson likelihood for age–sex–year-specific HAU-attributable death counts under a business-as-usual continuation of historical patterns (15). For each age–period stratum, let *Y*_*a,p*_ denote the number of HAU-attributable deaths and *N*_*a,p*_ the corresponding population size; the model assumes *Y*_*a*,*p*_ ∼ *Poisson*(μ_*a*,*p*_),


$$\log\,\left(\mu_{a,p}\right)=\log\,\left(N_{a,p}\right)+\eta_{a,p},$$

where η_*a*,*p*_ is an additive function of age, period and cohort effects. Thus, the BAPC model operates on the log-mortality-rate scale via a log link with a population offset, which is a standard formulation for age–period–cohort projections of count data. As an internal validation, the BAPC model was re-fitted using data for 1990–2010 only and then used to project HAU-attributable deaths and ASMRs for 2011–2021; these projections were compared with the corresponding GBD 2021 estimates, and predictive performance was summarized using RMSE, MAPE and correlation coefficients. Technical details of the priors, age–period–cohort identifiability constraints, handling of overdispersion, software implementation, convergence and model adequacy checks, and propagation of GBD uncertainty into projections are provided in Supplementary Method 1. The reported uncertainty reflects variation around this BAU path rather than unmodeled structural shocks. As an external benchmark, ARIMA models were fitted to the historical series (global and sex-specific). Model orders were selected by information criteria with standard diagnostics. All analyses were performed with R software (version 4.4.2).

## 3 Results

### 3.1 Global trends and population distribution of HAU exposure

**FIGURE 1:**
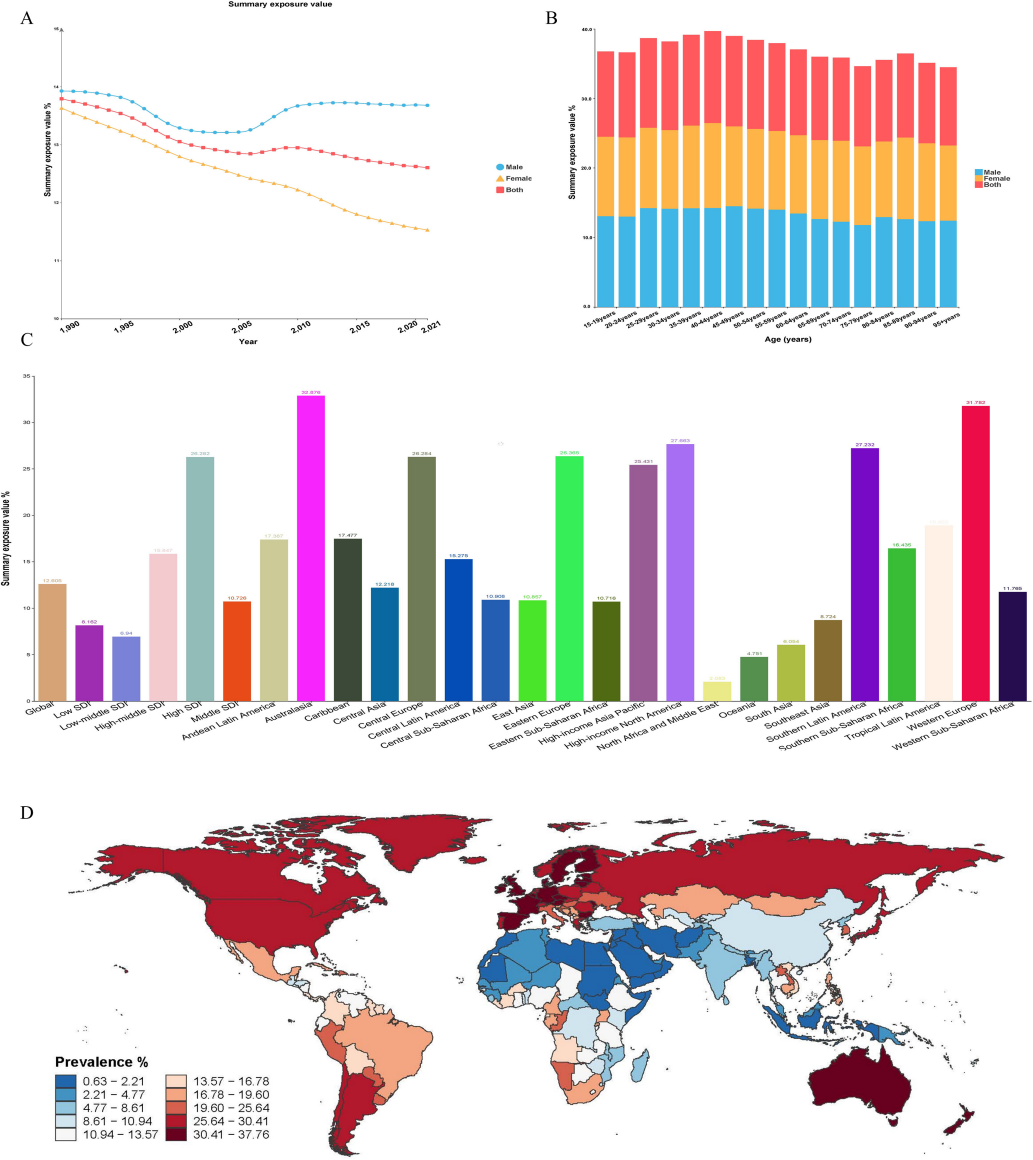
Global trends in HAU exposure, 1990–2021, by sex, age, and region. **(A)** SEV of HAU by sex. **(B)** SEV of HAU by age group. **(C)** SEV of HAU by region. **(D)** Prevalence of HAU by country. SEV, summary exposure value.

From 1990 to 2021, the global summary exposure value (SEV) for HAU declined overall (Figure 1A). SEV remained consistently higher in males than in females. For males, a modest rebound was seen after 2005, followed by a shallow plateau. In contrast, the female SEV fell steadily, reaching roughly 11% by 2021. Across all age groups, male SEVs were higher than female SEVs, and the largest sex differences were seen among adults aged 25–59 years. The population-level SEV peaked in the 40–44 years group at approximately 39.6%, and then decreased with increasing age (Figure 1B). Regions in the high SDI quintile exhibited markedly higher SEVs than those in middle and low SDI settings. Australasia showed the highest regional SEV (32.9%), followed by Western Europe (31.8%) and high-income North America (27.7 %) (Figure 1C). The prevalence of HAU in individual countries and territories is presented in Figure 1D.

### 3.2 Trends in the disease burden of CVD and T2DM caused by HAU from 1990 to 2021

**FIGURE 2:**
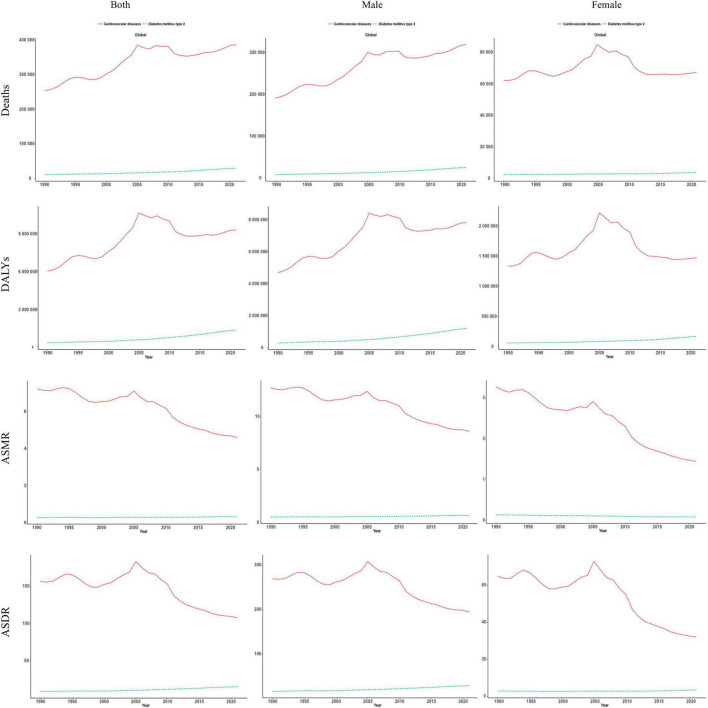
Global burden of CMDs attributable to HAU from 1990 to 2021. DALY, disability-adjusted life-year; ASMR, age-standardized mortality rate; ASDR, age-standardized DALY rate.

As shown in Figure 2, the number of HAU-attributable CVD deaths increased from approximately 253,062 (95% UI 82,603–492,246) in 1990 to 385,825 (95% UI 135,470–702,043) in 2021, a rise of 52.5 %. HAU-attributable DALYs due to CVD also increased from 6,014,463 (95% UI 1,525,791–11,305,117) to 9,276,924 (95% UI 2,899,413–16,424,137), an increase of approximately 54.2% (Supplementary Table 1). By contrast, HAU-attributable CVD ASMR and ASDR declined overall during 1990–2021, with ASMR decreasing from 7.23 to 4.60 per 100,000 and ASDR falling from 156.5 to 107.6 per 100,000, despite a transient peak around 2000–2005.

For T2DM, the HAU-attributable impact was more pronounced. Associated deaths rose from 10,215 in 1990 to 28,633 in 2021 (an increase of 180.3%), and DALYs from 0.34 million to 1.36 million (an increase of 304.5%) (Supplementary Table 2). HAU-attributable T2DM ASMR and ASDR increased steadily, with the increase slightly steeper in males than in females.

**FIGURE 3:**
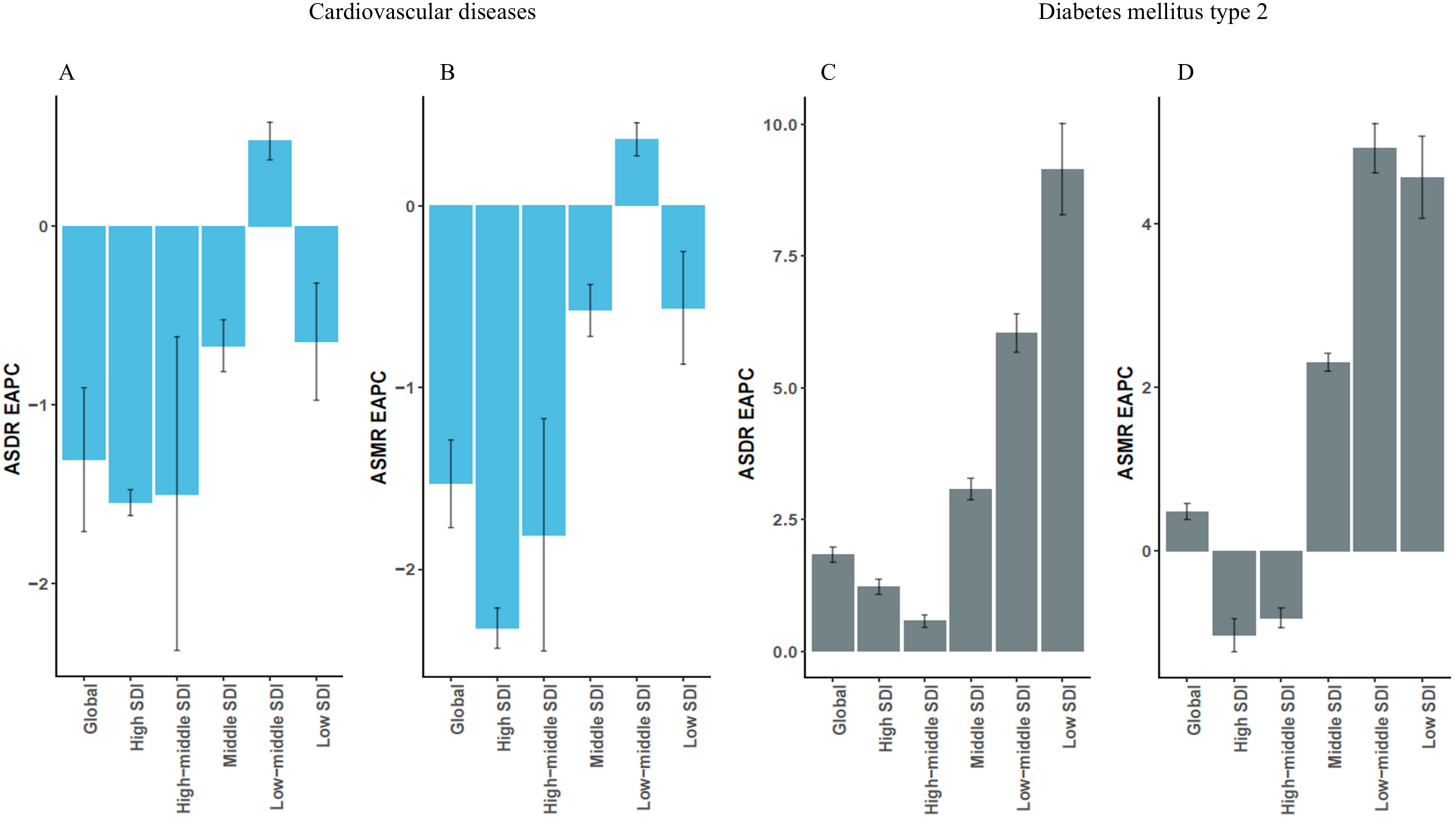
Estimated annual percentage changes (EAPCs) in the burden of CMDs attributable to HAU from 1990 to 2021. **(A,B)** EAPCs in ASMR and ASDR for alcohol-attributable CVD. **(C,D)** EAPCs in ASMR and ASDR for alcohol-attributable T2DM. EAPC, estimated annual percentage change; ASMR, age-standardized mortality rate; ASDR, age-standardized DALY rate.

From 1990 to 2021, EAPC summaries indicated that global HAU-attributable CVD ASMR and ASDR tended to fall (EAPC −1.53; 95% CI −1.77 to −1.29 and −1.31; 95% CI −1.71 to −0.90, respectively). The sharpest declines were seen in high-SDI and high-middle-SDI settings, while middle-SDI regions showed a small positive EAPC, which we interpret as suggesting some residual growth rather than a precisely estimated annual increase. In contrast, EAPCs for T2DM based on age-standardized rates suggested an overall upward tendency (ASMR EAPC 0.48; 95% CI 0.38 to 0.58 and ASDR 1.83; 95% CI 1.69 to 1.98) (Figures 3A, B). The largest increases were recorded in low- and low middle-SDI countries, while high-SDI regions changed little, and some showed small reductions in ASMR (Figures 3C, D).

### 3.3 Regional and SDI-level variations in the burden of CMDs attributable to HAU

Correlation analyses revealed distinct patterns in the relationship between the SDI and the burden of CMDs attributable to HAU. A non-linear, inverted U-shaped relationship was observed for CVD (Supplementary Figure 1A). HAU-attributable DALYs rose steadily across low- to middle-SDI settings (SDI ≈ 0.4–0.7), peaking around SDI 0.6, and then declined sharply in high SDI countries (SDI > 0.7). The peak of the curve was largely driven by Oceanian nations, which shows clear regional differences. In contrast, T2DM showed a clear positive gradient with SDI (Supplementary Figure 1B). HAU-attributable DALYs increased steadily with each SDI quintile, reaching their highest values in high-income regions–particularly High-income North America, High-income Asia Pacific, and Eastern Europe–where rates exceeded the global average by more than two-fold.

From 1990 to 2021, the number of CVD deaths attributable to HAU rose most sharply in high-middle SDI settings, climbing to about 210,000 in 2005 and then declining modestly after that, but remaining well above the 1990 baseline (Supplementary Figure 2A). For T2DM, HAU-related mortality increased steadily in every SDI quintile. The middle SDI group had the steepest increase after 2005, surpassing high-SDI regions by the mid-2010s, and the gap continued to widen after that (Supplementary Figure 2B).

Globally, the ASDR for CVD attributable to HAU showed a non-linear pattern over time, rising modestly until the early 2000s and then declining to a level slightly below that observed in 1990. The sharpest reduction occurred in high-middle SDI countries, where the ASDR fell by roughly 50% after peaking at nearly 500 per 100,000 in 2005. Conversely, the HAU-attributable ASDR for T2DM increased across all SDI regions, with the steepest increases seen in high- and high-middle-SDI settings. A similar pattern was seen for ASMR. CVD-related ASMR declined markedly in high-middle-SDI regions but rose in low-middle-SDI regions. For T2DM, the global HAU-attributable ASMR increased over time, with the largest increases again concentrated in high-middle-SDI countries after 2005, while other SDI groups had smaller increases (Supplementary Figure 3).

### 3.4 Regional and national differences in the burden of CMDs attributable to HAU

In 2021, Eastern Europe recorded the greatest cardiovascular burden attributable to HAU, with an ASDR of about 591 per 100,000 population, followed by Central Europe and Southern Sub-Saharan Africa (Supplementary Table 1). From 1990 to 2021, Southeast Asia showed the steepest annual increase in HAU-attributable CVD burden (ASMR EAPC 3.41%, 95% CI 3.08–3.74; ASDR 3.50%, 95% CI 3.17–3.83). For T2DM, the highest 2021 HAU-attributable ASDRs were observed in Southern Sub-Saharan Africa (≈ 48 per 100,000 population), Central Latin America and Oceania (Supplementary Table 2). Central Sub-Saharan Africa experienced the fastest escalation, with HAU-attributable ASMR and ASDR increasing by 9.58% and 26.38% per year, respectively.

**FIGURE 4:**
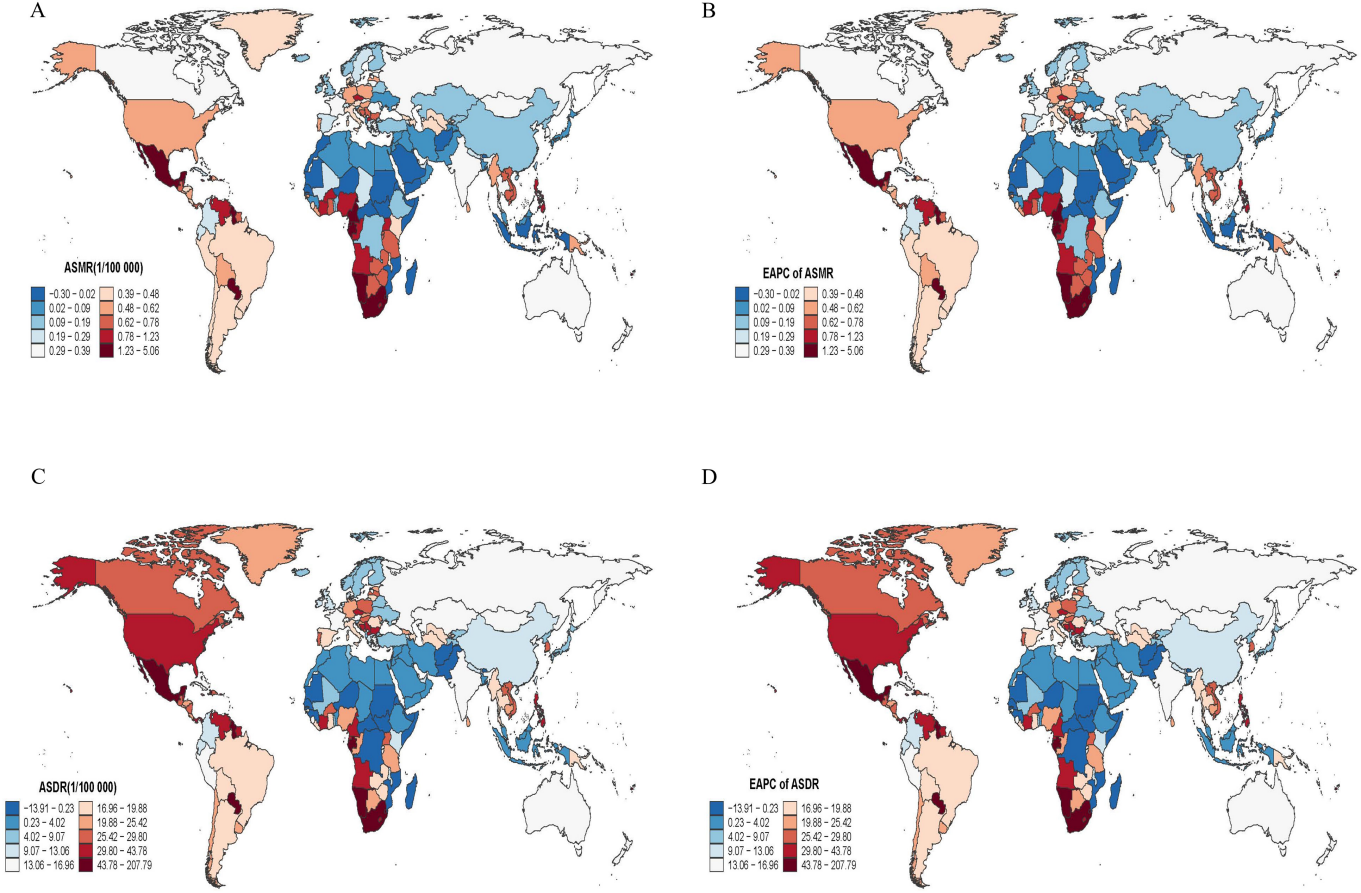
Global distribution and spatiotemporal trends of T2DM burden attributable to HAU. **(A)** ASMR of T2DM in 2021. **(B)** EAPC in ASMR from 1990 to 2021. **(C)** ASDR of T2DM in 2021. **(D)** EAPC in ASDR from 1990 to 2021. Country-specific 95% UIs for ASMRs and ASDRs, and 95% CIs for EAPCs, are reported in the Supplementary Tables and should be taken into account when interpreting color differences, especially for small or data-poor countries. EAPC, estimated annual percentage change; ASMR, age-standardized mortality rate; ASDR, age-standardized DALY rate.

Country-level analyses were broadly consistent with the regional and SDI patterns described above (Figure 4; Supplementary Figure 4 and Supplementary Tables 1, 2). In a subset of countries, particularly in parts of Eastern and Central Europe, Sub-Saharan Africa and Oceania, point estimates for EAPCs in HAU-attributable CVD and T2DM ASMR/ASDR were positive and comparatively large. However, in most of these locations the 95% CIs were wide and overlapped with those of many other countries, so the national estimates should be viewed as descriptive examples rather than precise rankings. Accordingly, the main inferences of this study are drawn at the SDI and regional level.

### 3.5 Sex- and age-specific differences in the burden of CMDs attributable to HAU

Supplementary Figure 5 shows that the ASMR and ASDR for CMDs attributable to HAU rise steadily with age and then decline in the oldest age groups. From 35 years onward, men consistently display higher CVD-related ASMRs than women, with a clear peak at 70–74 years. A sex differential in HAU-attributable CVD ASDR becomes evident in the 35–39-years band and reaches its maximum at 65–69 years. For T2DM, both sexes attain peak HAU-attributable ASMRs in the 70–74 years bracket, whereas the highest HAU-attributable ASDRs occur at 60–64 years. Across all ages, male HAU-attributable ASMR and ASDR values exceed those of females, showing that men bear a disproportionately large alcohol-attributable cardiometabolic burden.

**FIGURE 5:**
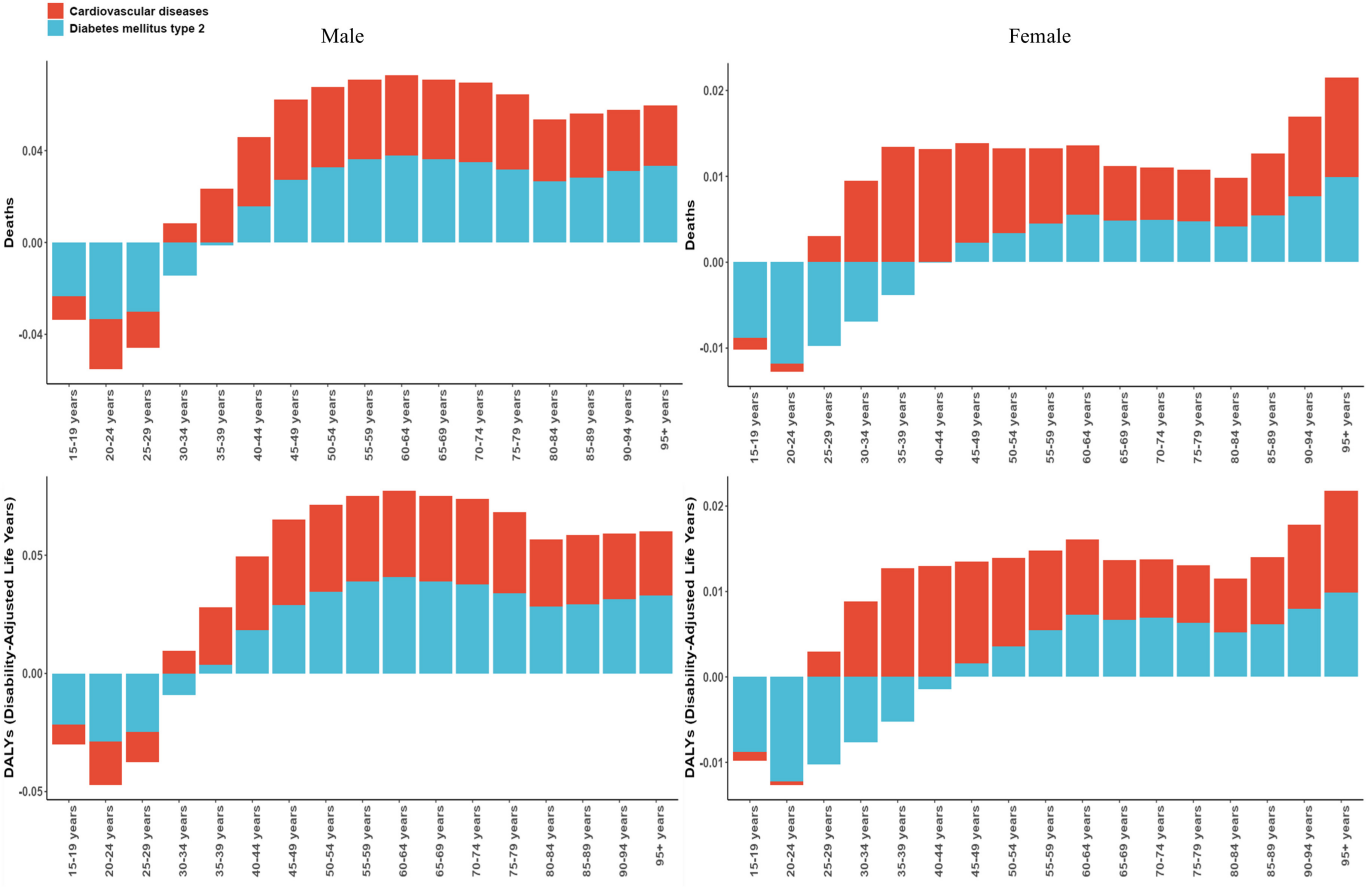
Sex- and age-specific analysis of mortality and DALYs attributable to HAU in CMDs in 2021.

In addition, Figure 5 illustrates marked differences in HAU-attributable mortality and DALYs burden attributable to HAU between CVD and T2DM across sex and age subgroups. Across all ages, CVD accounted for a larger share of deaths attributable to HAU than T2DM, with the disparity most striking in males. In mid-life (males 45–54 years; females 50–59 years), HAU-attributable T2DM DALYs approached, and occasionally exceeded, those of CVD. Among males, HAU-attributable deaths and DALYs peak at 65–69 years, then decline thereafter, and show a modest secondary rise at 85–89 years, although values remained above early-adult levels. In females, alcohol-related deaths and DALYs began to climb from 60 to 64 years onward and then increased steadily, reaching their highest levels in the ≥95-years group. These trajectories highlight a heavier alcohol-attributable cardiometabolic burden in men overall and an accelerating impact in very old adults.

In all 21 regions, men bore a larger alcohol-attributable burden than women. For CVD, the highest population-attributable fractions (PAFs) were recorded in Eastern Europe, Central Europe, and East Asia, each exceeding 5%. For T2DM, Central Europe ranked first, followed by Western Europe, High-income North America, and Australasia, with PAFs ranging from 5% to 8%. Even in low and low-middle SDI settings, where overall PAFs were below 2%, a clear male predominance persisted (Supplementary Figure 6).

### 3.6 Predicted burden of CMDs attributable to HAU from 2022 to 2040

**FIGURE 6:**
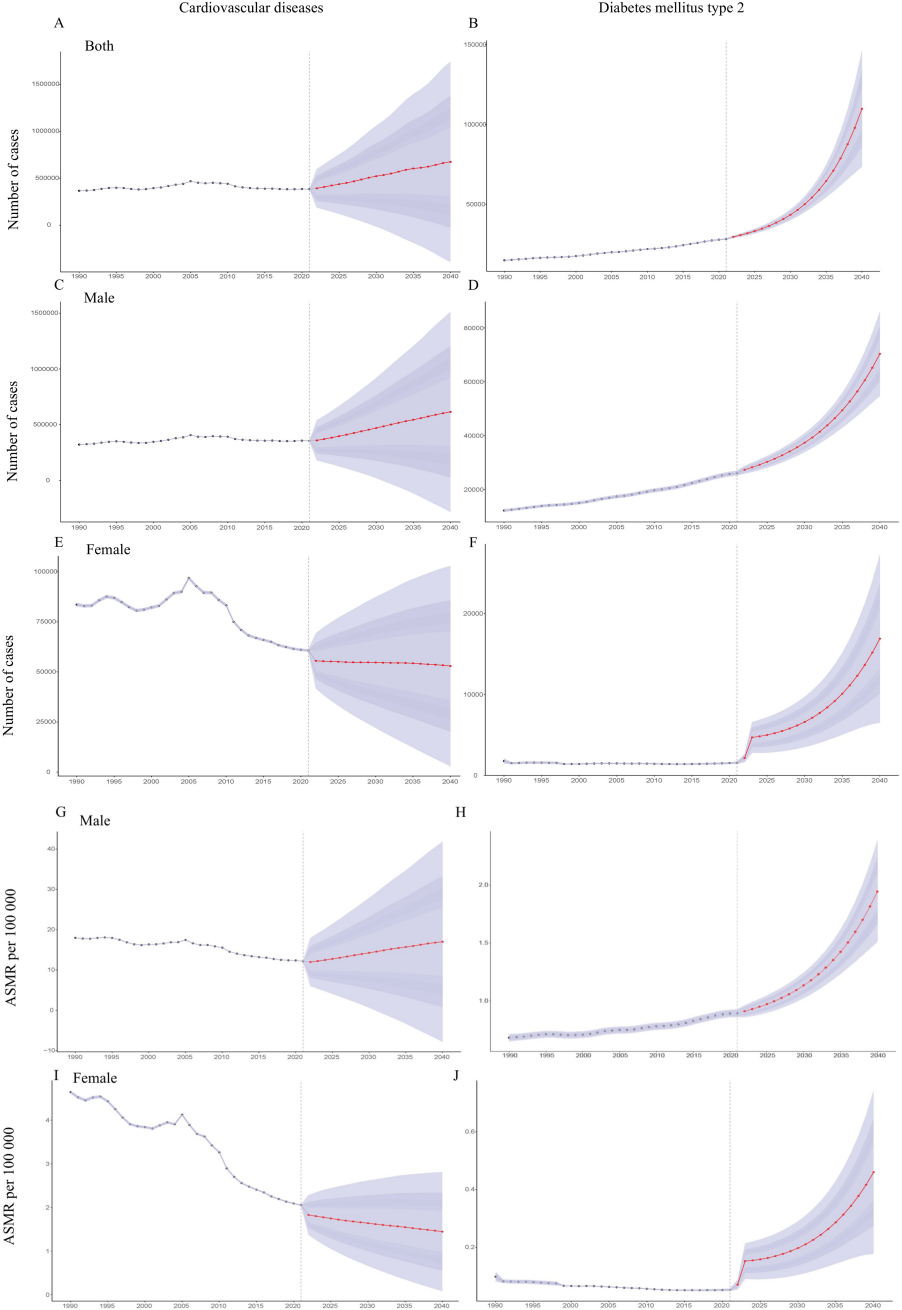
Projected global mortality and ASMRs for CMDs attributable to HAU (2022–2040). **(A–F)** Projected annual deaths by cause (CVD, T2DM) and sex. **(G–J)** projected ASMR by cause and sex. Shaded areas indicate 95% uncertainty intervals around the projected estimates, and the vertical dashed line marks the transition between historical estimates and projections. ASMR, age-standardized mortality rate.

Under a business-as-usual continuation of recent trends and policies, projections from the BAPC model indicate a sustained rise in deaths from CMDs attributable to HAU during 2022–2040 (Figures 6A–F). HAU-attributable cardiovascular deaths increased from 253,062 in 1990 to 385,825 in 2021 and, under a business-as-usual scenario, could reach around 675,000 by 2040 (an approximate 75% increase relative to 2021 in this scenario). Sex-specific projections indicate that male HAU-attributable CVD deaths are likely to rise markedly–approaching more than half a million by 2040–whereas female deaths are expected to decline slightly, thereby widening the gender gap. For T2DM, HAU-attributable deaths rose from 10,215 in 1990 to 28,633 in 2021 and, under the same business-as-usual scenario, could increase to around 110,000 by 2040 (roughly a three-fold increase). Most of this growth is expected to occur among men, with male deaths rising from the mid–20 000s to roughly 70,000, while female deaths may grow from only a few thousand to around 17,000. These trajectories suggest that T2DM will contribute disproportionately to future growth in the alcohol-related disease burden.

As shown in Figures 6G–J, the HAU-attributable ASMR for CVD is projected to continue increasing in males but to decline modestly in females over the projection period. In contrast, the HAU-attributable ASMR for T2DM is predicted to rise sharply in both sexes, showing the growing impact of HAU on CMDs mortality–particularly among men–over the next two decades. For comparison, ARIMA forecasts showed similar directions and magnitudes of change (Supplementary Figure 7). An internal truncated-data validation, in which BAPC was trained on 1990–2010 data and used to project 2011–2021, showed good agreement with GBD 2021 estimates for T2DM but larger discrepancies for CVD, especially in recent years (Supplementary Figure 8 and Supplementary Tables 3, 4). These results support using BAPC to describe long-term tendencies and scenario-based projections, while reinforcing that numerical values for 2040–particularly for CVD–should be viewed as approximate scenario ranges rather than precise predictions of future mortality rates or counts.

## 4 Discussion

Using GBD 2021 modeled estimates, we quantified the global burden of CMDs attributable to HAU from 1990 to 2021 and projected business-as-usual trends to 2040 using a BAPC model. In doing so, this secondary analysis complements previous GBD alcohol reports by focusing specifically on cardiometabolic diseases and jointly assessing CVD and T2DM within a single framework. Although overall HAU exposure has declined–particularly among women–both deaths and DALYs linked to CVD and T2DM have risen worldwide. T2DM DALYs have increased by more than 300% since 1990, and while CVD remains the largest absolute contributor, the metabolic impact of alcohol appears to be increasing in many regions.

Two main divergences need explanation: declining HAU exposure despite rising HAU-related mortality and DALYs, and decreasing CVD rates alongside increasing T2DM rates. First, average per capita intake has fallen, but binge and other high-risk drinking patterns persist and cause disproportionate harm (16). Second, CMDs have long latency periods, so current mortality largely reflects hazardous drinking that occurred decades earlier. Older adults are especially vulnerable because reduced metabolic capacity amplifies the adverse cardiovascular and metabolic effects of chronic HAU (17–19). Third, per capita alcohol consumption is increasing in many low-income countries while healthcare infrastructure remains limited, and enhanced diagnostic coding has improved case ascertainment (20–22). Together, these factors help explain the continuing rise in HAU-related deaths and DALYs, especially the steep growth observed for T2DM.

Alcohol exposure is consistently higher in men than in women across all age groups, with the largest gap between 40 and 79 years. In this age range, mortality and DALYs attributable to HAU peak, likely reflecting heavier intake, concurrent smoking, metabolic syndrome and occupational stress (23–25). According to the Global Status Report on Alcohol and Health, alcohol use is responsible for approximately 2.6 million deaths annually, accounting for 4.7 % of all global deaths, with the majority occurring in men (26). This male predominance is consistent with sex-specific biological differences in body-water fraction, alcohol-metabolizing enzyme activity in the stomach and liver, and hormonal milieu, together with differential responses in blood pressure, lipid handling, inflammation and insulin sensitivity. Sociocultural norms and occupational or peer contexts can further increase both opportunity and intensity of drinking among men. Behaviorally, men more often exhibit binge drinking and cluster alcohol with other risks such as tobacco use, unhealthy diet and lower uptake of preventive care. Taken together, these drivers align with the higher SEV and age-standardized rates observed in males. From an SDI perspective, the ASDR for CVD forms an inverted U shape, rising in low- and middle-SDI regions then declining in high-SDI settings. This pattern fits a sequence in which early economic development is accompanied by westernized lifestyles, wider alcohol availability and rapid urbanization, which may temporarily raise CVD burden, followed by later phases in which stronger regulation and better healthcare access contribute to lower mortality (27–29). In contrast, the ASDR for T2DM rises steadily with SDI and is highest in many high-income settings, where energy-dense diets, sedentary behavior and population aging often converge (30). The insidious onset and delayed diagnosis of diabetes may leave management gaps even where services are otherwise advanced (31). These interpretations are ecological and based on between-country associations, and should not be taken as direct evidence of the underlying mechanisms or of within-country socioeconomic gradients.

The ASMR and ASDR for CVD remain highest in Eastern Europe, Central Europe and Southern Sub-Saharan Africa, where heavy spirits consumption, binge-drinking norms and limited preventive services persist (32–34). A prospective multicenter cohort confirmed that alcohol intake in Eastern Europe is among the world’s highest and is dominated by strong spirits taken in binges, well-established triggers for cardiovascular events and premature mortality (35). In these settings, spirits-dominant, binge-prone use concentrates ethanol over short periods and aligns with acute hemodynamic, electrophysiological and pro-thrombotic pathways, which is consistent with persistently higher CVD burden. By contrast, the greatest alcohol-attributable burden of T2DM is observed in Southern Sub-Saharan Africa, Central Latin America and Oceania, which include many middle- and high-SDI contexts as well as some lower-SDI settings. Here, routine rather than episodic drinking is compounded by energy-dense diets and rising adiposity (36, 37). This combination has been hypothesized to promote chronic inflammation, ectopic lipid deposition and β-cell stress, which is consistent with the higher T2DM burden observed in these settings (38). Although some early observational studies reported apparently favorable associations for light-to-moderate drinking, these patterns are increasingly understood to reflect selection, misclassification and residual confounding rather than true benefit. Accumulating evidence indicates that even moderate, sustained intake can promote chronic inflammation, ectopic lipid deposition and oxidative stress and may accelerate both the onset and progression of diabetes (39, 40). Taken together, contemporary evidence does not support a cardiometabolic health benefit of low-dose alcohol consumption; any residual J-shaped associations in observational data are best interpreted as artifactual signals rather than genuine protective effects of alcohol itself. Finally, metabolic susceptibility, including the high prevalence of obesity and fatty liver and regional variation in alcohol-metabolizing capacity–may further lower harm thresholds at similar intakes. These mechanistic pathways are drawn from prior experimental and epidemiological literature and are presented here as hypotheses that fit the geographical patterns in our analysis, rather than mechanisms demonstrated by the GBD data themselves.

At the country level, patterns were heterogeneous. A small number of locations, including some small-population settings such as Israel, Iceland and Nepal and selected countries in Central and Sub-Saharan Africa and Oceania, showed comparatively large positive point estimates for EAPCs in HAU-attributable ASMR/ASDR (Supplementary Tables 1, 2). However, 95% CIs in these settings were generally wide and overlapped with those of many other countries, so these national estimates are used only illustratively, and our main inferences are anchored at the more stable SDI- and region-level patterns.

Under a business-as-usual continuation of recent trends and policies, scenario-based projections suggest that alcohol-attributable CVD deaths could increase on the order of 70%–80% between 2021 and 2040, and that T2DM deaths could approximately triple. These projections are inherently uncertain–especially the CVD estimates, for which truncated-data validation was less accurate than for T2DM–and unmodeled shocks such as major policy changes, economic crises or pandemics could materially alter their course. In a truncated-data validation, the BAPC model fitted to 1990–2010 data reproduced T2DM ASMR trends for 2011–2021 in broad terms, whereas deviations were larger for CVD, especially in recent years. This supports the use of BAPC to describe long-term tendencies and indicates that projections to 2040, particularly for CVD, should be viewed as approximate scenarios rather than precise forecasts of rates or counts. Despite this uncertainty, the projected increases are consistent with mechanistic evidence linking HAU to hypertension, dyslipidaemia and insulin resistance (41, 42), and to an elevated risk of premature death from diabetes complications (43, 44). Sex-stratified projections show sustained male predominance, whereas female CVD mortality may decline slightly, underscoring the need for male-focused strategies (45, 46). Because the projections assume BAU, stricter control measures such as higher excise taxes, tighter availability and marketing restrictions, and stronger enforcement would be expected to bend trajectories downward by reducing binge frequency and limiting per-occasion intake, whereas policy liberalization could shift them upward (47). At the metabolic level, HAU may interact with adiposity and fatty liver to increase insulin resistance, low-grade inflammation, oxidative stress, ectopic lipid deposition and β-cell stress, with possible contributions from chronic pancreatic injury (48–50). At the population level, aging and rising obesity, improved detection and coding of diabetes, and limitations in chronic-disease management can increase deaths in some settings (51, 52). These potential pathways, together with lagged effects from past drinking cohorts and declining cardiovascular mortality that reduces competing risks (53), are used here only to provide context for the projected trends and should not be interpreted as causal mechanisms demonstrated by this descriptive, model-based analysis. Overall, the projections are policy-contingent rather than deterministic and should be interpreted in light of fiscal space, enforcement capacity and primary-care systems.

Reported 95% UIs reflect variation propagated from exposure estimation, relative-risk inputs and PAF-based attribution, with additional uncertainty in BAPC projections. For trends, an EAPC whose 95% CI does not include zero indicates a clear direction; if the CI includes zero, the trend should be interpreted cautiously. For comparisons between locations, overlapping UIs call for conservative ranking, and wide UIs usually indicate sparse data. UIs around rates should be considered separately from those around counts, as demographic change can raise deaths or DALYs even when rates decline. Accordingly, PAF-based “attributable” burdens in this study should be interpreted as model-based scenario estimates within the GBD comparative risk assessment framework rather than as direct measures of individual-level causal effects. This interpretation assumes that the underlying relative risks are approximately causal and applicable to the study populations, and that residual confounding and selection, including the healthier profile of many moderate drinkers, may still be present.

These findings support the need for proactive and multifaceted alcohol control policies as part of broader cardiometabolic prevention strategies. Within the GBD comparative risk assessment, the PAFs used in this study describe the proportion of current CVD and T2DM burden that would be avoided under a hypothetical scenario where population exposure to HAU was reduced toward the theoretical minimum risk level. In practice, policy packages can only partly shift exposure distributions, so their real-world impact will be smaller and will depend on coverage, enforcement and behavioral responses. Core measures include higher excise taxes, tighter marketing restrictions, proportionate availability controls and sustained public education on alcohol harm. Pattern-sensitive strategies are needed where episodic heavy use is common, with priority actions to reduce binge frequency and limit per-occasion intake.

Regular cardiometabolic screening for high-risk drinkers, particularly middle-aged men, could facilitate earlier intervention. Gender-specific approaches for women in regions with rising trends and for men with high exposure are also important. High-SDI settings can link alcohol measures to existing diabetes and CVD programmes alongside taxation, availability and marketing controls; middle-SDI settings can focus on taxation and availability controls with primary-care integration; and low-SDI settings may need simple, high-yield measures such as excise taxes, restricted hours of sale and drink-driving enforcement, with opportunistic screening. Implementation should align with fiscal space, enforcement capacity and primary-care and data systems, and evaluation should focus on reducing binge episodes and increasing primary-care screening and brief-intervention coverage. Collaboration across health, education and finance sectors is essential to implement sustainable frameworks and curb the projected rise in alcohol-related CMDs.

Several limitations merit consideration. First, the GBD relies on self-reported alcohol consumption and represents population exposure by average consumption distributions. As a result, binge episodes may be under-captured and heterogeneity in drinking patterns is not fully represented, which can bias HAU-attributable estimates and precludes testing for a J-shaped relationship. Earlier J-shaped curves are now recognized to be partly driven by “sick quitter” bias–where former drinkers with poor health are grouped with abstainers–and by higher health consciousness and more favorable risk profiles among moderate drinkers. In line with this evidence, any apparent “protective” associations at low levels of intake are interpreted in this study as artifactual signals arising from selection and residual confounding, rather than as unresolved biological benefits of alcohol. In settings where light drinking predominates, limitations in exposure measurement and residual confounding in the underlying observational evidence may influence the CRA estimates, whereas in binge-prevalent settings the use of long-term mean intake without detailed pattern information may understate risk concentration. In addition, sparse primary data in some low-income settings necessitate borrowing, which can reduce precision. Although our analyses use GBD estimates across the full age range, CMD events in adolescents and young adults are rare and less consistently coded. In regions with substantial drinking among younger adults, our estimates may therefore still understate the full life-course cardiometabolic burden attributable to HAU. Second, the observational, population-level design entails residual confounding that cannot be fully excluded–including lifestyle clustering, comorbid behaviors and genetic predisposition–which may lead to under- or overestimation in specific subgroups. The mechanistic pathways discussed in this study are derived from prior experimental and epidemiological literature and are used as a contextual framework; they are not directly identified or tested in this descriptive, model-based analysis. SDI-based analyses are also inherently ecological: SDI is a composite country-level index, and associations between SDI and HAU-attributable burden describe between-country patterns rather than individual-level relationships. The GBD estimates analyzed here describe average patterns at the country, regional and SDI level; they do not distinguish differences by ancestry, cultural background, migration status or within-country socioeconomic position and may therefore not hold for specific subgroups without local data and validation. Third, attribution of CVD and T2DM to HAU uses population-attributable fractions within the GBD framework and is therefore attributional and association-based rather than an individual-level causal effect. These attributional estimates indicate the burden that could in principle be avoided under large shifts in exposure, but they should not be interpreted as direct predictions of the effect size that any specific alcohol policy package would achieve in practice. Accordingly, the reported HAU-attributable burdens should be interpreted as model-based scenario estimates under the GBD comparative risk assessment framework rather than definitive individual-level causal effects. The reported 95% uncertainty intervals propagate variability from both exposure estimation and relative-risk inputs, and adopting more conservative risk assumptions would lower absolute attributable magnitudes while preserving qualitative SDI-level trends. In addition, the relative risk functions underlying these estimates are derived from observational studies in which alcohol use often clusters with smoking, diet, physical inactivity, adiposity and socioeconomic disadvantage, so some unknown fraction of the estimated “HAU-attributable” burden may still reflect incomplete control for these correlated behaviors and social factors rather than alcohol alone. Fourth, EAPCs were derived from simple log-linear regressions of ASRs on calendar year, which do not propagate the full GBD uncertainty and may not fully capture non-linear trends; in this study they are therefore used as approximate descriptors of long-term tendencies, and small deviations around zero are interpreted as indicating roughly stable rates rather than strong evidence of change. CMDs in this study are defined narrowly as T2DM plus total CVD, and the CVD category aggregates multiple subtypes with different relationships to alcohol, so the results should not be taken as evidence of a single shared pathway from high alcohol use to all cardiometabolic conditions. Finally, although BAPC methods were used to forecast trends to 2040, projections remain sensitive to future changes in alcohol policy, cultural norms and consumption behavior.

## 5 Conclusion

Although population-level exposure to HAU has declined, HAU-attributable cardiometabolic mortality and DALYs continue to rise, with the sharpest increases occurring in men and in low and middle SDI settings. The marked geographic and socioeconomic heterogeneity observed in this study emphasizes the need for context-specific interventions. Projections suggest that, without intensified action, the alcohol-related chronic disease burden is likely to continue growing in the coming decades. Strengthening taxation and marketing restrictions, expanding health education and integrating alcohol control into chronic disease programmes may help to reduce HAU-related cardiometabolic burden, particularly in settings with high exposure and limited preventive capacity. The present findings may help guide more precise and sustainable strategies for alcohol reduction and CMDs prevention worldwide, while recognizing that they are derived from scenario-based analyses of GBD 2021 comparative risk assessment data.

## Data Availability

The original contributions presented in this study are included in this article/Supplementary material. Publicly available, de-identified data can be accessed at: https://vizhub.healthdata.org/gbd-results/. Further inquiries can be directed to the corresponding authors.

## Footnotes

1
https://vizhub.healthdata.org/gbd-results
