# Supplementary material for: Global burden and projections of cardiometabolic diseases attributable to high alcohol use: a comparative risk assessment based on the GBD 2021 study
Source: Front Nutr. 2026 Mar 6;13:1698730. doi: 10.3389/fnut.2026.1698730 (PMC13002442; doi:10.3389/fnut.2026.1698730)
Supplement: Supplementary file 1 [file Data_Sheet_1.docx]

# **Supplementary Method 1** BAPC projections

For each sex–location combination, age–period–cohort projections were generated using a BAPC model applied to annual **age-specific HAU-attributable death counts and population sizes** from GBD 2021. Let $Y_{a,p}$denote the number of HAU-attributable deaths in age group $a$and calendar year $p$, and let $N_{a,p}$denote the corresponding population size. We assumed
$Y_{a,p}\sim\text{Poisson}(\mu_{a,p})$,
$\log(\mu_{a,p})=\log(N_{a,p})+\alpha_{a}+\pi_{p}+\gamma_{c}$,
where $\alpha_{a}$, $\pi_{p}$and $\gamma_{c}$denote smooth age, period and cohort effects, respectively. This Poisson log-link formulation implies an additive structure on the log-mortality-rate scale and is standard for APC/BAPC modelling of rare-event count data. Priors on $\alpha_{a}$, $\pi_{p}$and $\gamma_{c}$, identifiability constraints and computational details are described below.

The model was implemented in R (version 4.4.2) using the BAPC package interfaced with INLA for approximate Bayesian inference. Model adequacy was evaluated using posterior predictive checks, comparison of fitted versus observed trends and sensitivity analyses to alternative hyperprior specifications. To propagate GBD uncertainty into the projections, the BAPC model was fitted across the full set of GBD posterior draws for age–sex–year-specific mortality rates. For each draw, predictive distributions for 2022–2040were obtained and then combined across draws to produce median projections and 95% uncertainty intervals. These projections represent a business-as-usual continuation of historical age, period and cohort patterns and do not endogenise future policy changes.

# **Supplementary Method 2** GBD 2021 risk–outcome specification and data extraction

**1.Risk factor and exposure definition**In the GBD comparative risk assessment, high alcohol use (HAU) is defined as consumption above the TMREL as specified by GBD. We did not impose a fixed TMREL ourselves; all attributable estimates were extracted directly from the GBD 2021 CRA outputs.

**2. Cause–risk pairs**In this study we analysed alcohol-attributable burden for two causes in the GBD 2021 cause hierarchy: “cardiovascular diseases” (total CVD) and “type 2 diabetes mellitus” (T2DM). For both causes, we used the alcohol-attributable estimates provided by the GBD 2021 comparative risk assessment for the risk factor “high alcohol use”. We did not analyse individual CVD subtypes separately.

**3. Relative risk functions and PAF formulation**
We did not re-estimate any relative risks. Instead, we used the standard GBD 2021 comparative risk assessment outputs, which are based on continuous, cause-specific dose–response functions for “high alcohol use” derived from meta-analyses of observational studies. For each age–sex–location–year stratum, GBD computes population-attributable fractions (PAFs) by comparing the observed exposure distribution with the TMREL. Alcohol-attributable deaths and DALYs are obtained by multiplying cause-specific deaths and DALYs by the corresponding PAFs. Our analysis uses these PAF-based alcohol-attributable deaths, DALYs and age-standardised rates exactly as provided by GBD 2021.

**4. GBD 2021 query settings and data extraction**Alcohol-attributable deaths, DALYs and age-standardised mortality and DALY rates for total CVD and T2DM were downloaded from the GBD Results Tool and the Global Health Data Exchange (GHDx). We extracted data for years 1990–2021, for all 204 countries and territories, both sexes, and age groups from 15–19 years to ≥95 years, consistent with the GBD 2021 comparative risk assessment for alcohol risk estimation, which provides exposure estimates for ages ≥15 years only. We selected the risk factor “high alcohol use” and the causes “cardiovascular diseases” and “type 2 diabetes mellitus”. Data were exported as comma-separated value (CSV) files and processed in R (version 4.4.2) to construct summaries by sex, age group, Sociodemographic Index (SDI) quintile, GBD region and country.

# **Supplementary Table 1** The global burden of Death and DALY due to CVD attributable to HAU in 1990 and 2021 and temporal trends from 1990 to 2021.

|  | 1990 | | 2021 | | 1990 | | 2021 | | EAPC | |
| --- | --- | --- | --- | --- | --- | --- | --- | --- | --- | --- |
| location | Deaths, (95% UI) | Age-standardized mortality rate, n × 10^-5^ (95% UI) | Deaths, (95% UI) | Age-standardized mortality rate, n × 10^-5^ (95% UI) | DALY, (95% UI) | Age-standardized DALY rate, n × 10^-5^ (95% UI) | DALY, (95% UI) | Age-standardized DALY rate, n × 10^-5^ (95% UI) | ASMR EAPC (95% CI) | ASDR EAPC (95% CI) |
| Global | 253061.89(82602.65,492245.68) | 7.23(2.43,14.03) | 385825.09(135470.10,702043.43) | 4.60(1.62,8.42) | 6014462.56(1525790.52,11305116.63) | 156.46(44.83,295.59) | 9276923.96(2899412.83,16424136.64) | 107.59(33.46,191.01) | -1.53(-1.77,-1.29) | -1.31(-1.71,-0.90) |
| High SDI | 65340.81(20145.95,132066.59) | 5.86(1.77,11.85) | 67004.11(25947.11,130121.97) | 2.83(1.05,5.42) | 1327384.24(271933.42,2690439.78) | 119.24(21.93,243.36) | 1481937.99(594322.49,2738277.87) | 72.11(26.99,131.94) | -2.32(-2.43,-2.21) | -1.55(-1.62,-1.48) |
| High-middle SDI | 112895.93(43494.56,208635.98) | 12.58(4.99,23.90) | 147629.02(68211.77,256194.13) | 7.68(3.60,13.24) | 2813800.39(1065978.99,5075002.03) | 286.47(110.05,515.56) | 3714828.91(1866693.88,6064980.32) | 196.03(100.33,317.67) | -1.81(-2.45,-1.17) | -1.50(-2.37,-0.62) |
| Middle SDI | 56166.32(11495.02,106844.23) | 6.24(1.57,11.74) | 126626.32(29388.70,233954.73) | 5.08(1.23,9.44) | 1405881.78(126241.45,2868697.27) | 139.87(24.54,269.79) | 2952364.57(475939.62,5554968.49) | 109.42(17.31,205.76) | -0.57(-0.72,-0.43) | -0.67(-0.81,-0.52) |
| Low-middle SDI | 10903.50(-1118.22,24518.27) | 2.08(0.03,4.56) | 30030.26(1877.46,65351.89) | 2.27(0.33,4.79) | 270083.53(-93976.51,660852.14) | 47.04(-7.13,107.94) | 761025.09(-71662.92,1733265.23) | 53.20(-0.06,116.50) | 0.37(0.28,0.46) | 0.48(0.37,0.58) |
| Low SDI | 7342.61(89.83,15562.00) | 3.80(0.47,7.82) | 14038.19(1586.36,30572.77) | 3.30(0.67,6.69) | 188649.55(-27396.81,422113.90) | 86.32(-0.55,182.94) | 355740.99(-17667.56,821480.80) | 73.80(7.64,159.90) | -0.56(-0.87,-0.25) | -0.64(-0.97,-0.32) |
| Andean Latin America | 248.89(-361.97,959.89) | 1.59(-1.30,4.94) | 494.74(-403.88,1587.27) | 0.92(-0.56,2.79) | 2569.81(-18098.09,24685.34) | 27.30(-56.68,120.44) | 6783.22(-21562.44,36456.34) | 14.32(-30.92,62.62) | -1.17(-1.55,-0.79) | -1.26(-1.83,-0.68) |
| Australasia | 1199.79(227.94,2543.38) | 5.28(1.02,11.18) | 1296.94(341.47,2748.69) | 2.05(0.47,4.36) | 21353.92(2168.82,48228.99) | 89.71(8.28,206.88) | 27141.83(7631.73,53226.68) | 47.45(8.38,94.66) | -2.88(-3.12,-2.64) | -1.70(-2.02,-1.38) |
| Caribbean | 850.93(52.44,2026.60) | 3.45(0.37,8.04) | 2455.48(1128.84,4245.14) | 4.52(2.05,7.84) | 20652.17(-3555.54,52799.26) | 82.91(-4.17,202.51) | 63179.14(26628.28,109717.06) | 116.98(48.07,204.24) | 1.45(1.26,1.63) | 1.71(1.50,1.92) |
| Central Asia | 1845.79(73.06,4649.80) | 4.24(0.62,10.39) | 3969.79(1517.01,7708.18) | 5.24(2.08,10.29) | 47699.13(-10552.11,127094.09) | 104.84(-11.03,265.40) | 112705.98(38212.52,216743.81) | 131.82(47.66,250.89) | 0.79(0.36,1.22) | 0.79(0.31,1.28) |
| Central Europe | 20725.60(6439.78,42714.04) | 14.94(4.73,30.93) | 22591.77(10625.27,40201.29) | 9.79(4.82,17.19) | 432954.16(92053.06,890632.22) | 293.22(61.81,611.46) | 473221.02(224510.69,806440.72) | 219.26(105.05,365.08) | -1.43(-1.55,-1.30) | -0.99(-1.13,-0.85) |
| Central Latin America | 1164.88(-715.88,3346.90) | 1.91(-0.26,4.70) | 1696.68(-1062.45,5000.72) | 0.73(-0.36,2.13) | 15418.80(-45246.90,76895.14) | 32.75(-29.32,99.63) | 21943.18(-70661.30,123765.69) | 9.74(-25.21,49.23) | -3.73(-3.96,-3.50) | -5.02(-5.41,-4.64) |
| Central Sub-Saharan Africa | 1040.36(-232.56,2569.40) | 6.31(0.23,13.97) | 2325.39(-98.78,5303.07) | 5.80(0.68,12.08) | 24483.53(-13235.11,66987.80) | 128.65(-25.24,306.21) | 55793.01(-15305.47,142166.24) | 119.26(0.23,264.69) | 0.19(-0.54,0.92) | 0.30(-0.44,1.05) |
| East Asia | 82166.61(19836.62,153160.50) | 11.23(3.03,20.73) | 152424.43(36745.63,295700.53) | 7.42(1.79,14.34) | 2069754.70(360404.80,3977273.94) | 244.44(52.33,458.03) | 3461297.94(699035.15,6645195.67) | 158.57(29.13,307.50) | -1.28(-1.40,-1.16) | -1.31(-1.45,-1.18) |
| Eastern Europe | 42623.99(23687.44,75380.19) | 16.17(9.27,28.60) | 54300.17(39151.69,78059.34) | 17.20(12.77,23.98) | 1251427.61(726213.47,2035926.37) | 464.45(277.21,747.24) | 1745074.52(1324916.52,2320030.80) | 590.80(456.40,764.36) | -0.30(-1.69,1.10) | 0.23(-1.33,1.81) |
| Eastern Sub-Saharan Africa | 4153.62(485.56,8457.33) | 6.57(1.38,12.77) | 7306.25(961.11,14874.20) | 5.26(1.23,10.28) | 108440.54(-178.80,234110.48) | 148.97(17.80,303.18) | 186497.08(2581.27,399933.24) | 116.82(17.31,237.01) | -0.92(-1.18,-0.66) | -1.03(-1.31,-0.75) |
| High-income Asia Pacific | 12879.99(1479.79,26372.43) | 7.10(0.85,14.48) | 10607.81(223.07,23536.54) | 1.70(-0.09,3.83) | 299531.95(22031.00,599295.48) | 151.92(12.89,303.59) | 217640.95(12733.49,460453.77) | 45.13(-2.89,96.98) | -5.11(-5.40,-4.81) | -4.38(-4.71,-4.05) |
| High-income North America | 10862.63(4302.97,20249.95) | 2.97(1.10,5.55) | 19296.64(9362.61,35145.53) | 2.85(1.42,5.16) | 244734.41(89158.23,442054.06) | 69.71(23.07,125.25) | 494283.82(253152.76,840531.95) | 81.16(39.89,136.97) | -0.05(-0.29,0.19) | 0.75(0.60,0.89) |
| North Africa and Middle East | 1175.72(240.76,2472.07) | 0.81(0.23,1.63) | 1714.97(518.04,3394.78) | 0.45(0.15,0.88) | 31034.88(575.83,71664.24) | 18.61(3.00,39.55) | 43556.81(8403.99,93111.25) | 9.74(2.53,19.69) | -2.18(-2.39,-1.97) | -2.40(-2.60,-2.20) |
| Oceania | 55.50(-13.03,146.18) | 2.12(-0.14,5.37) | 90.21(-42.28,269.00) | 1.38(-0.35,3.72) | 1645.14(-731.47,4679.39) | 53.62(-10.43,139.03) | 2634.56(-1878.58,8662.58) | 34.19(-14.40,98.86) | -1.19(-1.66,-0.73) | -1.26(-1.74,-0.78) |
| South Asia | 3585.53(-5226.00,13460.43) | 0.80(-0.66,2.57) | 16318.58(-6003.58,44404.66) | 1.24(-0.30,3.21) | 66307.36(-211855.84,358781.71) | 16.26(-25.71,62.02) | 364456.43(-288254.39,1133717.98) | 26.49(-14.51,75.58) | 1.76(1.22,2.31) | 2.05(1.28,2.82) |
| Southeast Asia | 4420.87(-896.63,10298.38) | 2.00(-0.07,4.41) | 29976.76(7003.65,56514.29) | 4.89(1.15,9.16) | 110265.49(-69672.18,299390.61) | 47.18(-12.39,110.96) | 808263.56(127797.76,1564490.09) | 118.32(21.80,226.80) | 3.41(3.08,3.74) | 3.50(3.17,3.83) |
| Southern Latin America | 4192.82(1079.41,8186.12) | 9.55(2.57,18.63) | 2784.13(516.15,5653.38) | 3.01(0.47,6.19) | 97730.87(18349.49,192292.38) | 213.62(42.34,417.35) | 53390.36(-1107.76,115144.18) | 58.65(-5.21,131.50) | -3.43(-3.59,-3.27) | -4.12(-4.27,-3.97) |
| Southern Sub-Saharan Africa | 1626.73(442.89,3008.31) | 6.94(2.42,12.52) | 3729.76(1364.66,6573.50) | 7.51(3.02,12.91) | 40479.04(4590.72,81408.77) | 155.54(33.92,294.37) | 91338.55(27330.53,167112.34) | 163.19(56.92,291.14) | 0.12(-0.35,0.59) | -0.01(-0.44,0.42) |
| Tropical Latin America | 6019.17(1792.62,11097.99) | 6.90(2.11,13.16) | 6354.67(1162.76,12813.67) | 2.53(0.48,5.08) | 172750.36(49324.58,320266.67) | 176.39(51.93,323.25) | 144436.83(4366.44,311872.96) | 55.53(1.73,119.99) | -3.42(-3.69,-3.16) | -4.13(-4.43,-3.82) |
| Western Europe | 46563.62(11992.68,98430.85) | 7.61(1.90,16.09) | 35038.54(12782.56,67465.14) | 2.85(0.88,5.55) | 810387.36(153231.23,1724227.05) | 132.45(14.71,289.48) | 610742.33(201561.72,1158606.14) | 57.46(13.41,111.48) | -3.13(-3.25,-3.01) | -2.70(-2.79,-2.60) |
| Western Sub-Saharan Africa | 5658.86(1136.90,11166.86) | 7.43(1.79,14.63) | 11051.38(2705.93,20741.03) | 6.72(1.66,12.70) | 144841.31(20874.60,283762.36) | 167.65(31.48,326.71) | 292542.81(60406.32,550745.42) | 150.12(38.21,280.26) | -0.52(-0.66,-0.38) | -0.56(-0.71,-0.40) |
| Note: Country-specific EAPCs are reported with 95% CIs derived from log-linear regressions fitted to GBD central estimates of age-standardised rates; these CIs reflect regression uncertainty only and do not propagate GBD posterior uncertainty. By contrast, GBD-derived burden estimates (deaths, DALYs, ASMR/ASDR) and projection outputs are reported with 95% UIs. Apparent ‘top’ or ‘bottom’ rankings are statistically unstable and often overlap; interpret them as descriptive summaries rather than precise league tables. | | | | | | | | | | |

# **Supplementary Table 2** The global burden of Death and DALY due to T2DM attributable to HAU in 1990 and 2021 and temporal trends from 1990 to 2021.

|  | 1990 | | 2021 | | 1990 | | 2021 | | EAPC | |
| --- | --- | --- | --- | --- | --- | --- | --- | --- | --- | --- |
| location | Deaths, (95% UI) | Age-standardized mortality rate, n × 10^-5^ (95% UI) | Deaths, (95% UI) | Age-standardized mortality rate, n × 10^-5^ (95% UI) | DALY, (95% UI) | Age-standardized DALY rate, n × 10^-5^ (95% UI) | DALY, (95% UI) | Age-standardized DALY rate, n × 10^-5^ (95% UI) | ASMR EAPC (95% CI) | ASDR EAPC (95% CI) |
| Global | 10215.25(3342.14,19579.54) | 0.29(0.10,0.54) | 28632.54(10094.25,54905.26) | 0.34(0.12,0.64) | 336146.88(22954.74,740575.94) | 8.89(1.29,18.74) | 1359606.17(338151.83,2834915.44) | 15.43(3.67,32.25) | 0.48(0.38,0.58) | 1.83(1.69,1.98) |
| High SDI | 4891.50(1800.32,8993.08) | 0.43(0.16,0.80) | 7704.95(2770.32,14384.98) | 0.34(0.12,0.65) | 161659.19(38227.53,324773.23) | 14.58(3.09,29.54) | 446525.05(90148.58,946319.98) | 22.03(2.31,50.62) | -1.03(-1.23,-0.83) | 1.23(1.09,1.36) |
| High-middle SDI | 2960.05(1116.32,5339.19) | 0.32(0.12,0.58) | 5084.67(2011.19,9332.11) | 0.26(0.10,0.47) | 106742.69(26710.90,209686.50) | 10.79(2.77,20.97) | 269068.68(61971.53,559650.94) | 13.16(2.58,28.12) | -0.81(-0.93,-0.70) | 0.58(0.46,0.69) |
| Middle SDI | 1765.37(342.14,3721.30) | 0.19(0.05,0.39) | 10110.32(3741.67,18563.58) | 0.38(0.14,0.70) | 57480.72(-14099.09,156819.75) | 6.12(0.23,14.42) | 440747.75(138482.87,893000.76) | 15.50(4.88,31.45) | 2.31(2.20,2.42) | 3.08(2.87,3.28) |
| Low-middle SDI | 465.52(-207.93,1426.06) | 0.09(-0.02,0.26) | 4512.18(1439.06,9088.73) | 0.33(0.11,0.67) | 10933.26(-20769.57,51483.09) | 2.31(-2.12,8.37) | 168894.76(27777.73,389650.55) | 11.58(2.50,25.65) | 4.92(4.62,5.23) | 6.04(5.68,6.40) |
| Low SDI | 112.66(-502.92,968.08) | 0.09(-0.18,0.47) | 1179.54(-26.27,3088.86) | 0.29(0.03,0.67) | -1446.41(-25521.73,31594.15) | 0.88(-8.21,13.33) | 32472.69(-24932.11,124957.52) | 7.99(-1.52,23.03) | 4.57(4.07,5.07) | 9.15(8.28,10.02) |
| Andean Latin America | 17.34(-36.17,87.03) | 0.11(-0.14,0.45) | 228.74(12.74,515.15) | 0.40(0.03,0.89) | 6.14(-2613.42,3176.14) | 1.77(-9.33,15.65) | 8299.02(-2334.35,22382.25) | 14.55(-2.49,37.57) | 5.47(5.05,5.89) | 9.47(8.62,10.32) |
| Australasia | 128.87(50.31,232.98) | 0.55(0.21,0.99) | 227.60(61.27,450.32) | 0.38(0.09,0.76) | 4190.05(1131.28,7693.93) | 17.66(4.45,33.05) | 8899.64(245.56,21542.20) | 15.65(-1.34,40.33) | -1.59(-1.94,-1.24) | -0.73(-0.91,-0.56) |
| Caribbean | 167.58(46.13,337.03) | 0.66(0.19,1.33) | 363.87(112.99,685.53) | 0.67(0.21,1.26) | 5583.19(841.85,12239.32) | 22.43(4.38,47.65) | 17591.64(3726.04,36724.86) | 32.12(6.61,67.83) | -0.06(-0.16,0.05) | 1.07(0.97,1.16) |
| Central Asia | 57.25(14.19,117.72) | 0.12(0.03,0.25) | 273.54(94.40,523.18) | 0.32(0.11,0.60) | 2616.24(-5.61,5880.78) | 5.49(0.30,11.89) | 15163.60(3184.52,30777.97) | 16.47(3.94,32.85) | 2.88(2.44,3.32) | 3.28(3.01,3.56) |
| Central Europe | 605.10(220.19,1073.41) | 0.40(0.14,0.71) | 1268.39(492.26,2336.69) | 0.54(0.20,1.02) | 28154.52(7711.26,55493.18) | 18.15(4.95,36.31) | 61704.07(17251.14,127779.73) | 28.31(6.31,60.22) | 1.31(0.98,1.65) | 1.63(1.42,1.84) |
| Central Latin America | 615.96(91.84,1292.40) | 0.80(0.18,1.61) | 2951.20(926.77,5441.45) | 1.17(0.38,2.15) | 19039.34(-5349.72,51133.95) | 25.96(0.09,60.35) | 111548.46(21440.06,219280.26) | 42.99(8.48,84.04) | 0.79(0.46,1.13) | 1.15(0.83,1.47) |
| Central Sub-Saharan Africa | -13.17(-182.58,192.25) | 0.05(-0.69,0.98) | 146.61(-165.35,644.45) | 0.40(-0.17,1.33) | -1991.73(-8379.92,5822.90) | -3.29(-26.89,26.52) | 783.44(-15131.84,24082.31) | 8.00(-13.67,41.84) | 9.58(5.85,13.44) | 26.38(17.62,35.80) |
| East Asia | 697.07(119.37,1489.26) | 0.09(0.03,0.18) | 3254.99(1283.94,6109.12) | 0.15(0.06,0.29) | 27314.25(-21389.11,84451.01) | 3.47(-1.20,9.15) | 214487.12(59908.39,443772.10) | 9.24(2.06,20.52) | 2.04(1.73,2.34) | 3.48(2.81,4.15) |
| Eastern Europe | 224.36(74.26,408.17) | 0.08(0.03,0.14) | 902.28(336.93,1629.71) | 0.24(0.09,0.46) | 14632.34(2730.06,30242.42) | 4.89(0.38,10.30) | 48129.60(11121.68,99959.69) | 13.13(1.61,28.68) | 2.63(1.70,3.56) | 2.70(2.33,3.06) |
| Eastern Sub-Saharan Africa | 13.43(-401.89,541.32) | 0.09(-0.43,0.78) | 460.10(-195.30,1581.59) | 0.36(-0.06,1.01) | -2269.73(-17701.90,16604.58) | -0.18(-17.09,21.36) | 9009.29(-19996.74,54111.46) | 8.18(-5.20,30.32) | 4.45(4.25,4.65) | 16.86(14.40,19.38) |
| High-income Asia Pacific | 379.39(56.30,848.03) | 0.19(0.03,0.42) | 457.79(121.80,974.34) | 0.09(0.02,0.21) | 16594.02(-8169.90,46068.43) | 7.88(-3.93,22.18) | 45124.83(-5525.31,126758.35) | 10.58(-6.22,37.54) | -2.64(-2.85,-2.42) | 0.36(-0.16,0.89) |
| High-income North America | 1556.57(566.21,2877.39) | 0.44(0.14,0.81) | 3329.99(1188.71,5992.25) | 0.50(0.17,0.91) | 60444.17(12489.98,117420.28) | 18.10(3.69,35.94) | 236042.71(57891.15,450876.93) | 37.04(7.55,74.61) | -0.08(-0.49,0.33) | 2.27(2.12,2.42) |
| North Africa and Middle East | 88.92(29.41,179.44) | 0.06(0.02,0.11) | 294.96(115.51,537.77) | 0.07(0.03,0.12) | 3583.22(970.91,7355.87) | 2.02(0.61,4.06) | 18607.76(5763.98,35753.30) | 3.73(1.23,7.14) | 0.70(0.48,0.92) | 1.99(1.82,2.15) |
| Oceania | 18.61(4.01,42.71) | 0.62(0.14,1.37) | 57.52(16.30,111.08) | 0.75(0.23,1.45) | 633.62(82.51,1543.13) | 19.23(3.82,43.70) | 2295.80(422.46,4847.68) | 26.47(6.40,53.27) | 0.96(0.64,1.28) | 1.37(1.06,1.69) |
| South Asia | 336.03(-213.21,1138.30) | 0.07(-0.03,0.21) | 4346.32(1311.69,8597.47) | 0.32(0.10,0.61) | 9411.23(-20103.56,47567.02) | 1.87(-2.37,7.53) | 180052.16(35743.17,410422.02) | 11.75(2.58,25.95) | 6.11(5.43,6.79) | 7.10(6.35,7.85) |
| Southeast Asia | 156.91(-100.66,564.82) | 0.07(-0.02,0.22) | 2356.00(897.17,4652.38) | 0.37(0.14,0.72) | 4533.50(-7892.55,22373.35) | 2.20(-1.72,8.48) | 97485.33(30614.61,196502.03) | 13.99(4.85,27.73) | 6.37(6.07,6.68) | 6.98(6.70,7.27) |
| Southern Latin America | 404.41(146.63,756.47) | 0.88(0.32,1.65) | 415.86(108.06,868.48) | 0.46(0.12,0.97) | 12252.02(3432.60,23794.94) | 26.10(7.32,50.70) | 18227.46(1451.11,43519.12) | 20.55(0.80,50.04) | -2.08(-2.23,-1.94) | -0.80(-0.95,-0.66) |
| Southern Sub-Saharan Africa | 232.09(65.18,482.38) | 0.95(0.29,1.96) | 915.08(308.19,1793.03) | 1.73(0.62,3.29) | 6278.78(1079.63,14588.65) | 24.71(5.43,55.39) | 27159.18(7642.58,54602.67) | 47.59(14.51,94.26) | 1.98(1.59,2.36) | 2.12(1.83,2.42) |
| Tropical Latin America | 340.84(78.73,687.51) | 0.41(0.11,0.79) | 1296.52(378.39,2542.95) | 0.51(0.15,0.99) | 12117.33(667.41,29012.98) | 14.01(2.41,30.54) | 52367.02(9261.72,115352.68) | 19.80(3.55,43.53) | 0.72(0.31,1.12) | 1.10(0.75,1.45) |
| Western Europe | 3961.51(1541.61,7087.34) | 0.65(0.25,1.16) | 3944.26(1308.55,7441.47) | 0.36(0.11,0.68) | 107892.68(27054.96,209395.21) | 18.02(3.60,35.96) | 152704.56(13535.85,354091.81) | 15.38(-2.41,42.56) | -2.09(-2.24,-1.94) | -0.84(-1.02,-0.66) |
| Western Sub-Saharan Africa | 226.18(-21.13,670.16) | 0.32(0.00,0.85) | 1140.94(315.59,2437.53) | 0.73(0.25,1.51) | 5131.71(-4280.25,19904.99) | 7.15(-2.37,23.76) | 33923.48(1780.19,82424.75) | 19.54(4.29,43.26) | 2.74(2.58,2.90) | 3.27(3.11,3.43) |
| Note: Country-specific EAPCs are reported with 95% CIs derived from log-linear regressions fitted to GBD central estimates of age-standardised rates; these CIs reflect regression uncertainty only and do not propagate GBD posterior uncertainty. By contrast, GBD-derived burden estimates (deaths, DALYs, ASMR/ASDR) and projection outputs are reported with 95% UIs. Apparent ‘top’ or ‘bottom’ rankings are statistically unstable and often overlap; interpret them as descriptive summaries rather than precise league tables. | | | | | | | | | | |

# **
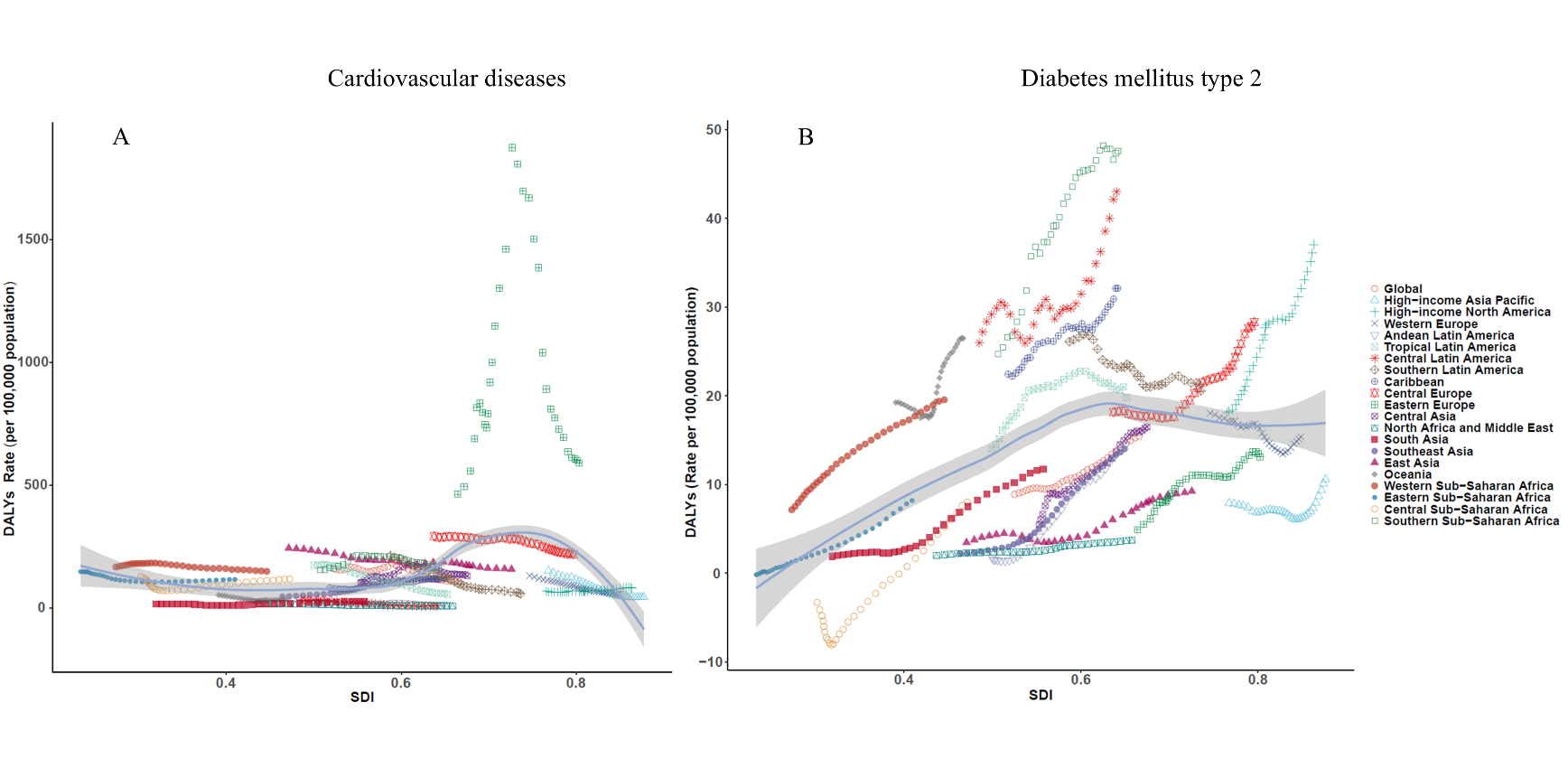
Supplementary Figure 1** Relationship between SDI and CMDs burden attributable to high alcohol use

# **Supplementary Figure 2** Trends in mortality due to alcohol-related cardiometabolic diseases across global and SDI regions from 1990 to 2021


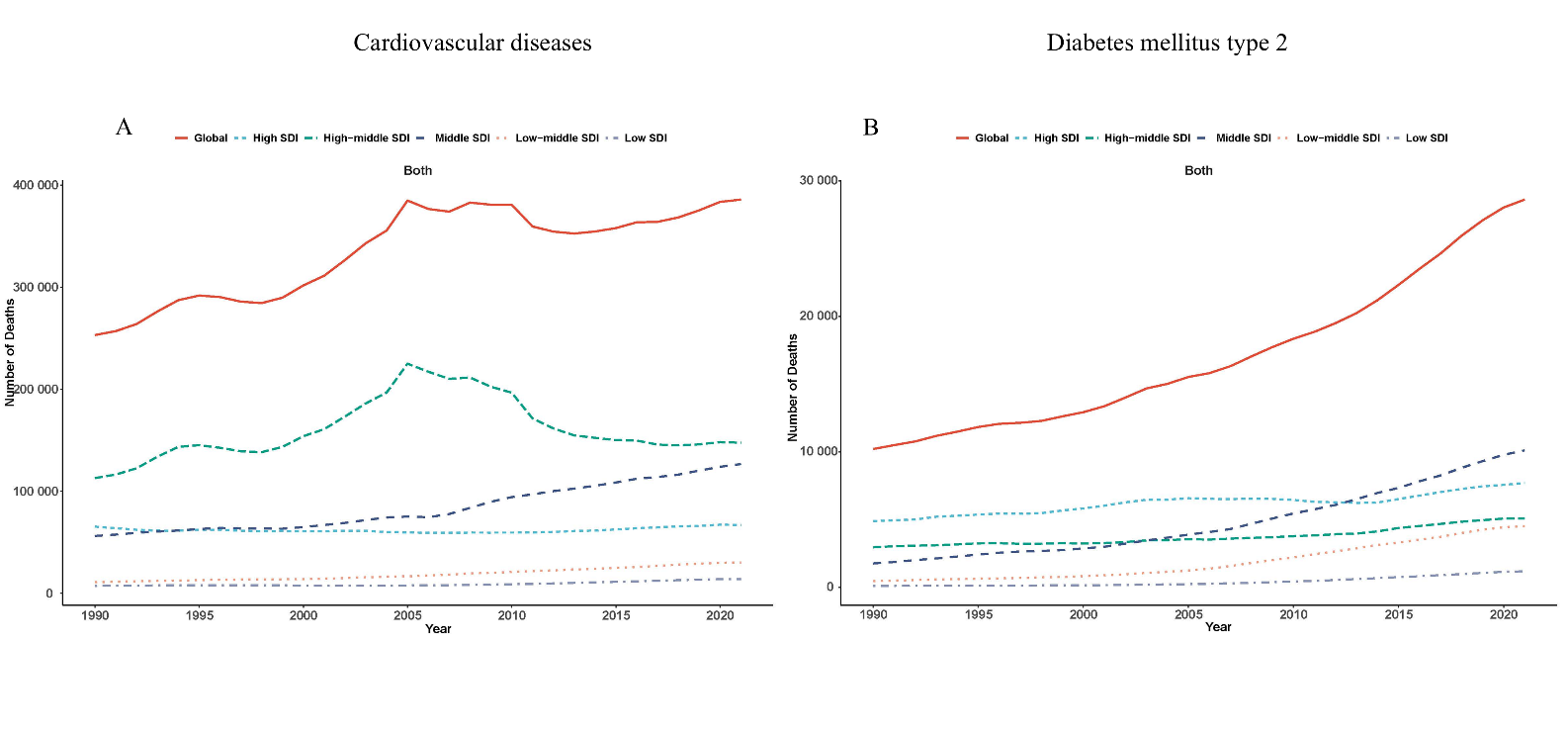


# **Supplementary Figure 3** Trends in ASMR and ASDR of alcohol-related CMDs across global and SDI regions, 1990–2021.
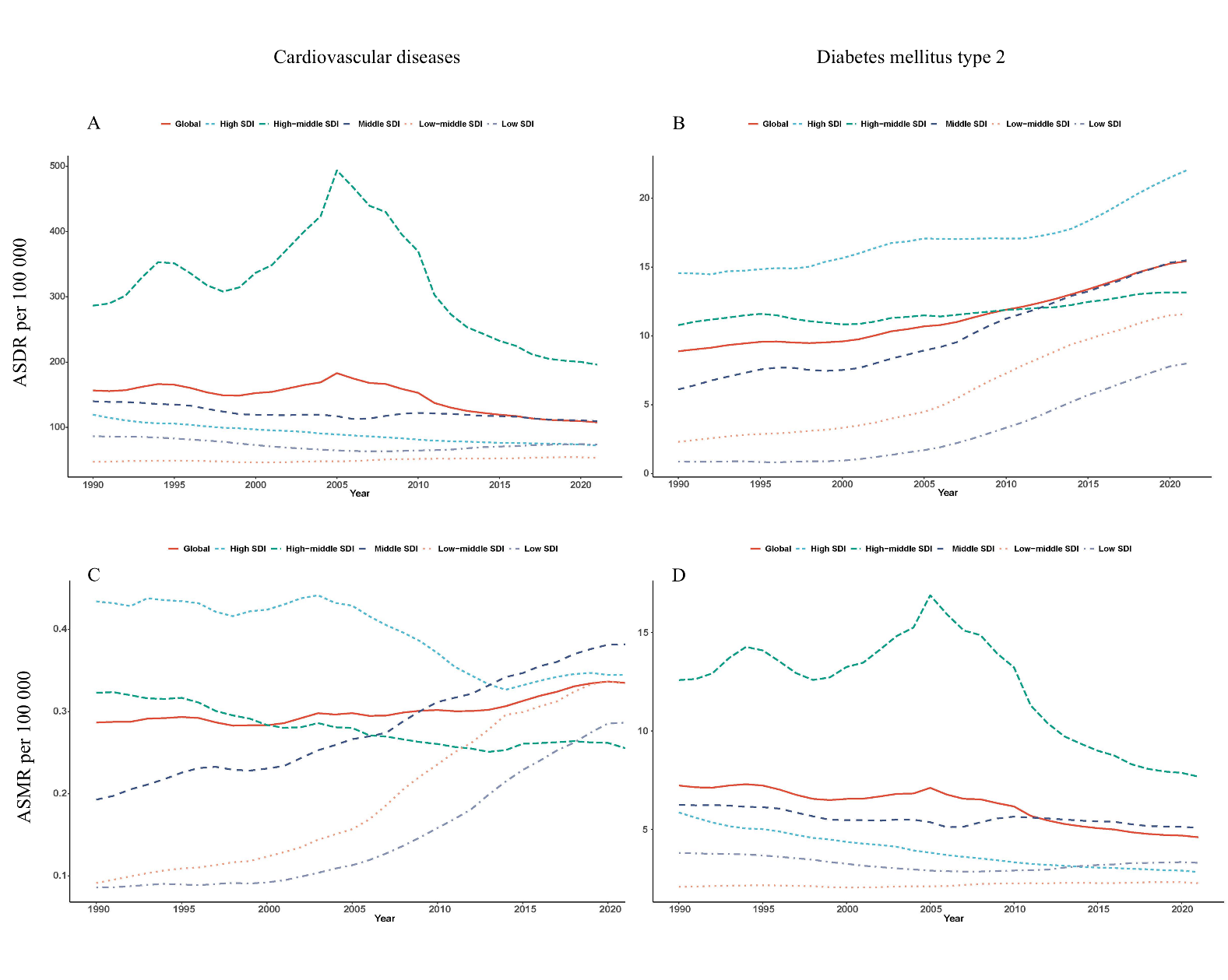


#
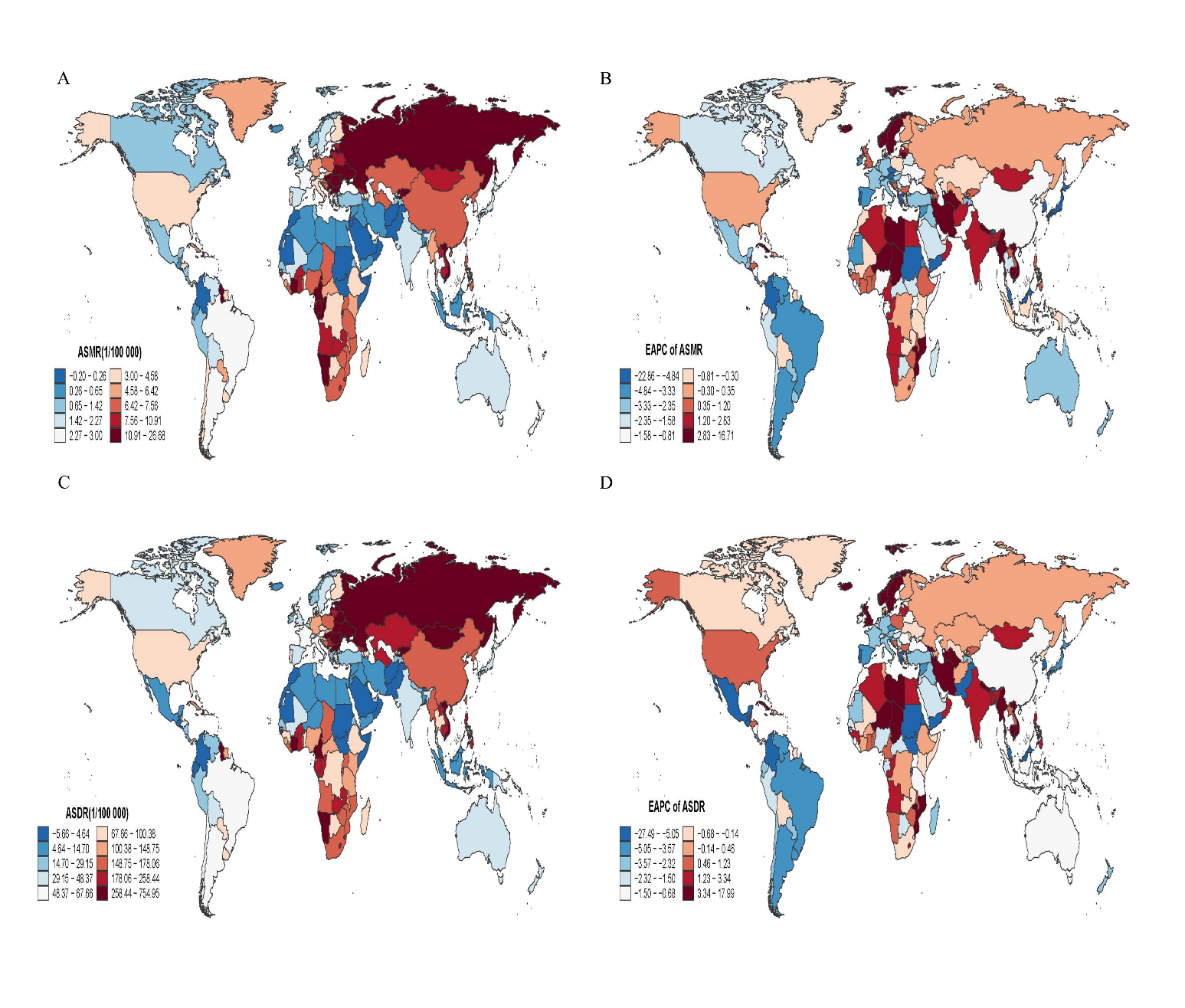
**Supplementary Figure 4** Global distribution and spatiotemporal trends of the burden of CVD attributable to HAU.

# **Supplementary Figure 5** Age- and sex-specific patterns of CMDs burden attributable to high alcohol use


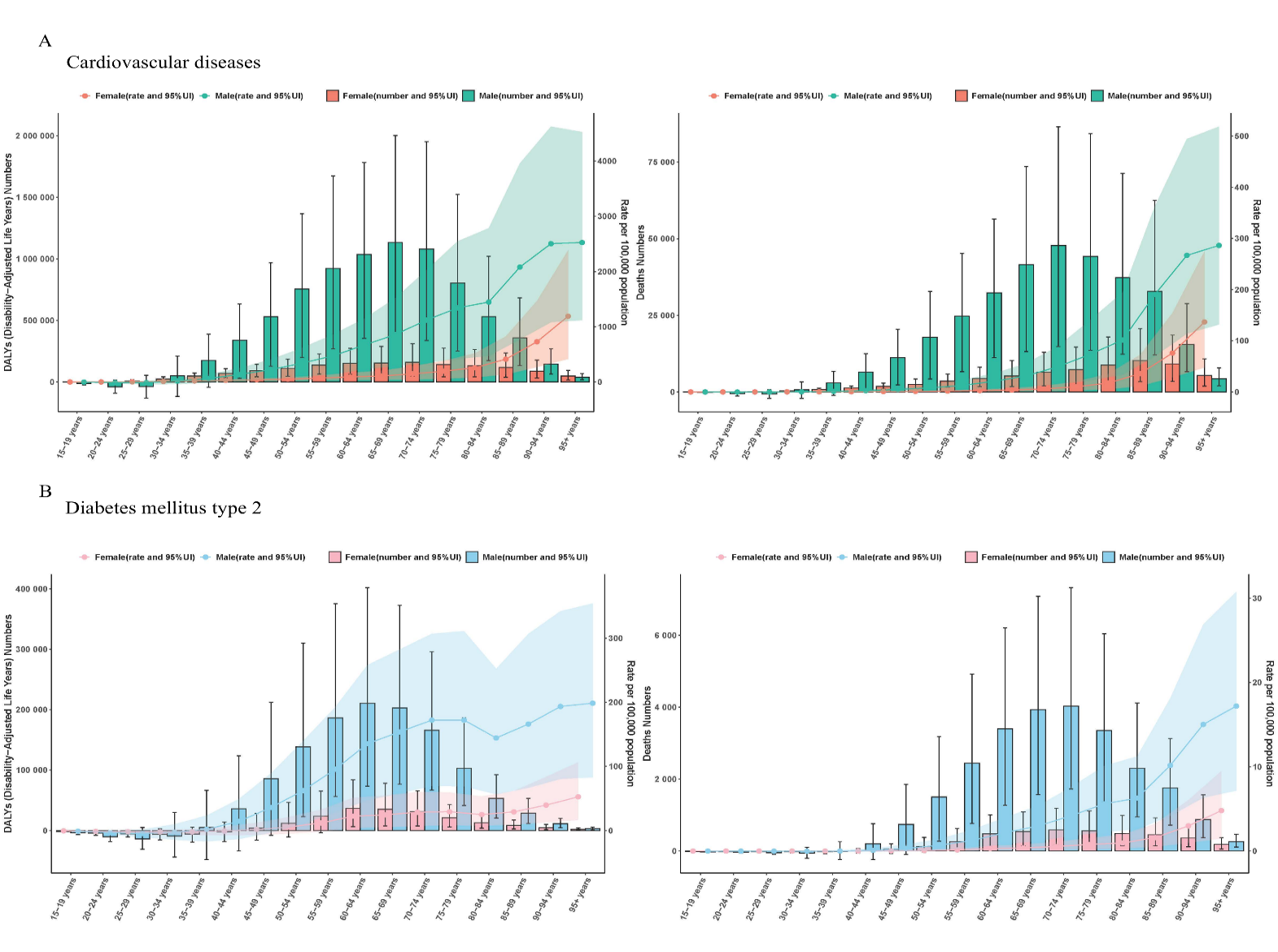


# **Supplementary Figure 6** Regional and sex-specific patterns of mortality from CMDs attributable to high alcohol use worldwide


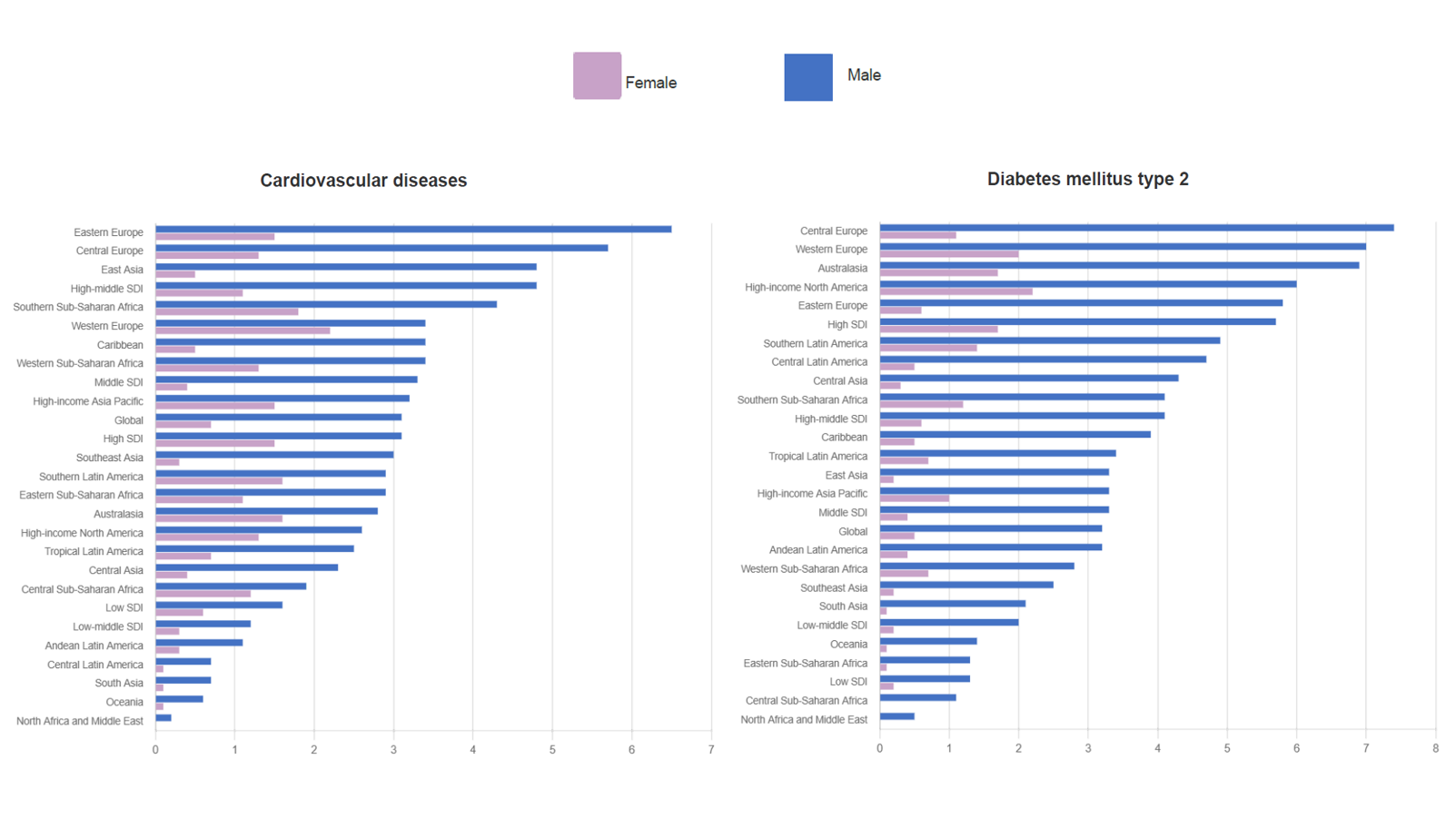


# **Supplementary Figure 7 ARIMA forecasts for alcohol-attributable CVD and T2DM mortality (global and by sex), 1990–2040.**


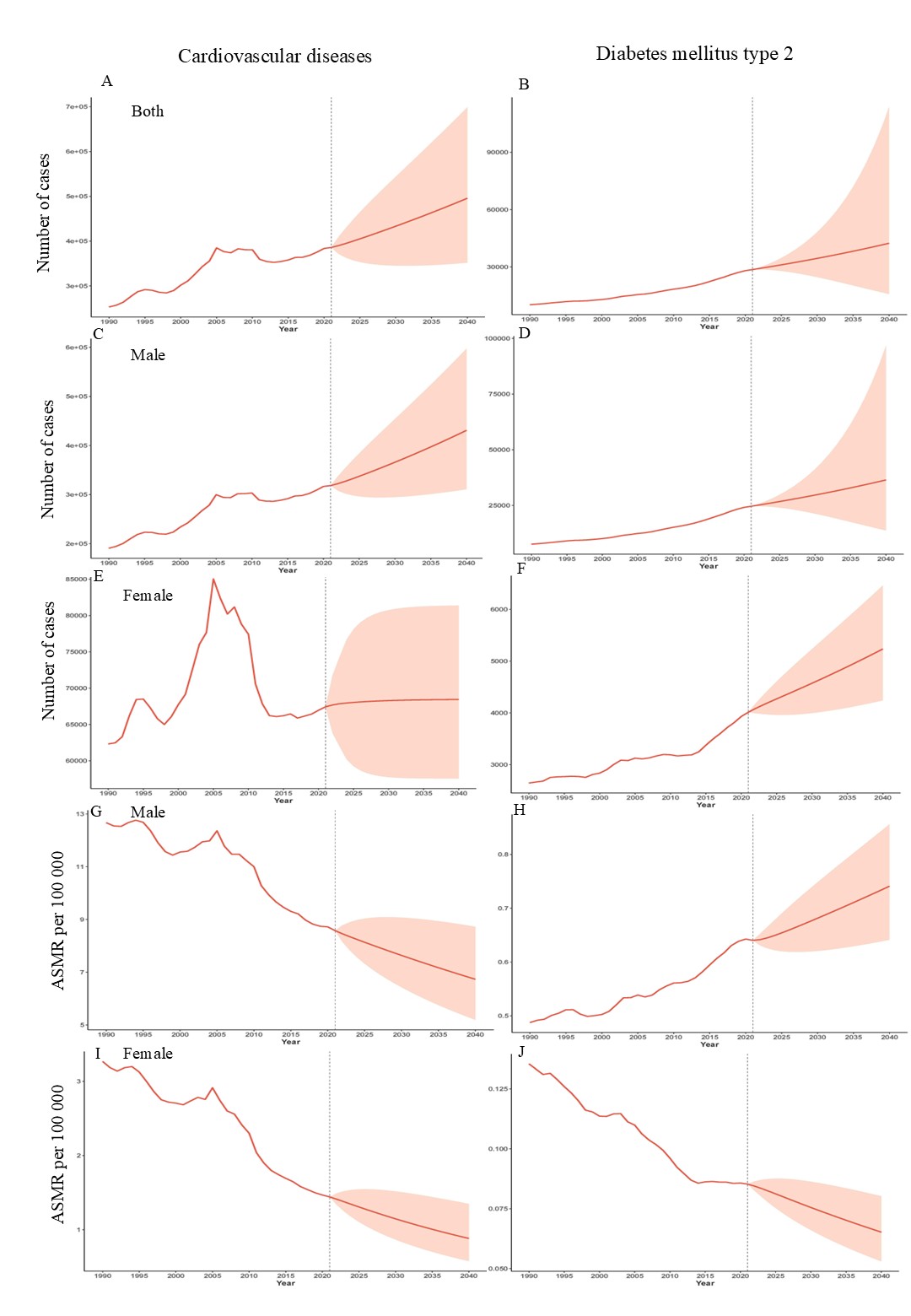


# **Supplementary Figure 8.** Observed and BAPC-projected global HAU-attributable ASMRs for CVD and T2DM, 1990–2021 (train 1990–2010; validation 2011–2021).


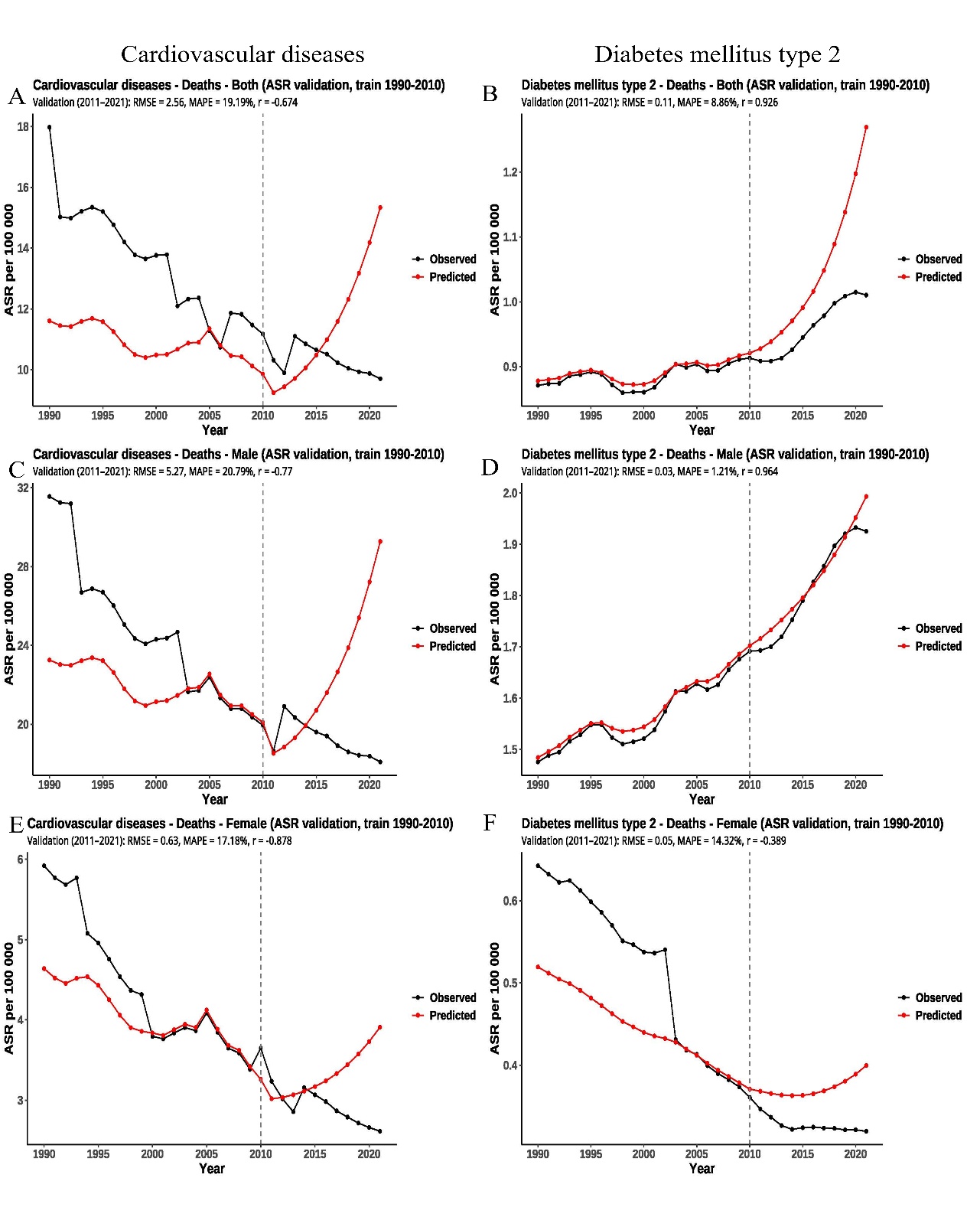


# **Supplementary Table 3**. Truncated-data validation of BAPC projections based on HAU-attributable ASMRs, 2011–2021 (train 1990–2010).

| **Cause** | **Sex** | **Validation period** | **RMSE (ASMR)** | **MAPE (%)** | **Correlation (r)** |
| --- | --- | --- | --- | --- | --- |
| CVD | Both | 2011–2021 | 2.56 | 19.19 | −0.674 |
| CVD | Male | 2011–2021 | 5.27 | 20.79 | −0.770 |
| CVD | Female | 2011–2021 | 0.63 | 17.18 | −0.878 |
| T2DM | Both | 2011–2021 | 0.11 | 8.86 | 0.926 |
| T2DM | Male | 2011–2021 | 0.03 | 1.21 | 0.964 |
| T2DM | Female | 2011–2021 | 0.05 | 14.32 | −0.389 |

# **Supplementary Table 4.** Truncated-data validation of BAPC projections based on HAU-attributable deaths, 2011–2021 (train 1990–2010).

| **Cause** | **Sex** | **Validation period** | **RMSE**  **(number of deaths)** | **MAPE (%)** | **Correlation (r)** |
| --- | --- | --- | --- | --- | --- |
| CVD | Both | 2011–2021 | 231 000 | 54.46 | 0.967 |
| CVD | Male | 2011–2021 | 202 000 | 59.83 | 0.986 |
| CVD | Female | 2011–2021 | 28 600 | 38.73 | −0.247 |
| T2DM | Both | 2011–2021 | 4 210 | 15.94 | 0.981 |
| T2DM | Male | 2011–2021 | 3 320 | 16.89 | 0.996 |
| T2DM | Female | 2011–2021 | 565 | 14.86 | 0.991 |

# **Supplementary Code S1. Calculation of estimated annual percentage change (EAPC)**

For each stratum defined by cause, sex and location, temporal trends in (ASRs were summarised using the EAPC. Let $\text{ASR}_{t}$denote the age-standardised rate in calendar year $t$. A simple log-linear regression model was fitted:

$$ln(\text{ASR}_{t})=\alpha+\beta\times t+\varepsilon_{t},$$

where $\alpha$is the intercept, $\beta$is the slope with respect to calendar year, and $\varepsilon_{t}$is the error term. The EAPC and its 95% confidence interval (CI) were derived from the slope parameter $\beta$as:

$$\text{EAPC}=100\times[exp(\beta)-1].$$

The 95% CI for the EAPC was obtained by applying the same transformation to the lower and upper bounds of the 95% CI for $\beta$. In the main text, a positive EAPC and CI entirely above zero were interpreted as indicating an increasing trend, a negative EAPC and CI entirely below zero as a decreasing trend, and a CI including zero as indicating that rates were approximately stable over time.

EAPCs were computed in R (version 4.4.2). A template function used in the analysis is shown below.

| ## Calculation of EAPC  compute_eapc <- function(year, asr) {  df <- data.frame(year = year, asr = asr)  # remove missing or non-positive ASR values (log requires >0)  df <- df[is.finite(df$year) & is.finite(df$asr) & df$asr > 0, ]  # ensure at least 3 time points  if (nrow(df) < 3L) {  return(data.frame(EAPC = NA, EAPC_low = NA, EAPC_high = NA, n = nrow(df)))  }  fit <- stats::lm(log(asr) ~ year, data = df)  beta <- unname(stats::coef(fit)[["year"]])  # use t critical value (more appropriate than 1.96 for small n)  se_beta <- summary(fit)$coefficients["year", "Std. Error"]  t_crit <- stats::qt(0.975, df = fit$df.residual)  eapc <- 100 * (exp(beta) - 1)  eapc_low <- 100 * (exp(beta - t_crit * se_beta) - 1)  eapc_high <- 100 * (exp(beta + t_crit * se_beta) - 1)  data.frame(  EAPC = eapc,  EAPC_low = eapc_low,  EAPC_high = eapc_high,  n = nrow(df)  )  } |
| --- |

# **Supplementary Code S2. Bayesian age–period–cohort (BAPC) projection model**

For projections of HAU-attributable deaths and ASMRs, a BAPC model was used. Age-specific death counts were arranged in an age-by-period matrix for each cause and sex. Let $Y_{a,p}$denote the number of HAU-attributable deaths in age group $a$and period $p$, and $N_{a,p}$the corresponding population. A Poisson likelihood with a log link and an offset for population size was assumed:

$$Y_{a,p}\sim\text{Poisson}(\mu_{a,p}),\log(\mu_{a,p})=\log(N_{a,p})+\eta_{a,p},$$

where

$$\eta_{a,p}=\alpha+f_{\text{age}}(a)+f_{\text{period}}(p)+f_{\text{cohort}}(c),$$

and the cohort index $c$is defined by the usual identity $c=p-a$. Smooth age, period and cohort effects were modelled as second-order random walks (RW2) with weakly informative priors on the precision parameters. Identifiability was handled by centring the age, period and cohort effects to have mean zero.

The model was fitted in R (version 4.4.2) using the **BAPC** package as an interface to **INLA**. Historical data for 1990–2021 were used to estimate the posterior distributions of the APC effects. Projections for 2022–2040 were obtained by extending the period dimension and sampling from the posterior predictive distribution under a business-as-usual continuation of past trends. Posterior means and 95% credible intervals for fitted and projected death counts were then combined with standard population weights to derive historical and projected ASMRs.

A simplified template for the BAPC implementation is shown below.

| ## Bayesian age–period–cohort (BAPC) projection model  ## Package: BAPC (INLA-based). Model: Poisson likelihood for counts with population person-years.  ## Required packages  ## install.packages("BAPC", repos = "https://R-Forge.R-project.org")  ## install.packages("INLA",  ## repos = c(getOption("repos"),  ## INLA = "https://inla.r-inla-download.org/R/stable"))  library(INLA)  library(BAPC)  ## -----------------------------  ## 1) Prepare input data (IMPORTANT: rows = periods/years; cols = age groups)  ## -----------------------------  ## deaths_df: data.frame, n_period x n_age  ## pop_df : data.frame, same dim as deaths_df  ## Columns should correspond to 5-year age groups: 15–19, 20–24, ..., ≥95  ## Rows should correspond to calendar years: 1990, 1991, ..., 2021, 2022, ..., 2040  ##  ## For FUTURE years (2022–2040):  ## - deaths_df must be NA (to be projected)  ## - pop_df must be provided (GBD/UN population projections)  years_hist <- 1990:2021  years_fut <- 2022:2040  years_all <- c(years_hist, years_fut)  age_labels <- c("15-19","20-24","25-29","30-34","35-39","40-44","45-49","50-54",  "55-59","60-64","65-69","70-74","75-79","80-84","85-89","90-94","≥95")  ## Example placeholders (replace with your study data):  ## deaths_hist: matrix/data.frame with rows = years_hist, cols = age_labels  ## pop_all : matrix/data.frame with rows = years_all, cols = age_labels  ##  ## deaths_hist <- ...  ## pop_all <- ...  ## Build deaths_df with NA rows for future years  deaths_df <- as.data.frame(deaths_hist)  stopifnot(all(rownames(deaths_df) %in% as.character(years_hist)) \|\| nrow(deaths_df) == length(years_hist))  ## Ensure correct dim and colnames  colnames(deaths_df) <- age_labels  rownames(deaths_df) <- as.character(years_hist)  deaths_future_na <- matrix(NA, nrow = length(years_fut), ncol = length(age_labels),  dimnames = list(as.character(years_fut), age_labels))  deaths_df <- rbind(deaths_df, deaths_future_na)  ## Population (person-years) must cover historical + future years  pop_df <- as.data.frame(pop_all)  colnames(pop_df) <- age_labels  rownames(pop_df) <- as.character(years_all)  ## -----------------------------  ## 2) Create APCList object  ## -----------------------------  ## gf = 5 because age groups are 5-year bands and periods are 1-year intervals  apc_obj <- APCList(epi = deaths_df, pyrs = pop_df, gf = 5,  agelab = age_labels, periodlab = as.character(years_all))  ## -----------------------------  ## 3) Fit BAPC model and project  ## -----------------------------  n_proj <- length(years_fut)  fit <- BAPC(  apc_obj,  predict = list(npredict = n_proj, retro = FALSE),  model = list(  age = list(model = "rw2", prior = "loggamma", param = c(1, 0.00005)),  period = list(include = TRUE, model = "rw2", prior = "loggamma", param = c(1, 0.00005)),  cohort = list(include = TRUE, model = "rw2", prior = "loggamma", param = c(1, 0.00005)),  overdis= list(include = TRUE, model = "iid", prior = "loggamma", param = c(1, 0.005))  ),  ## stdweight: vector length = number of age groups; will be normalised internally  ## stdweight = std_pop,  verbose = FALSE  )  ## Add quantiles / prediction intervals (optional but recommended)  fit <- qapc(fit, percentiles = c(0.025, 0.5, 0.975))  ## -----------------------------  ## 4) Extract projected deaths (counts) and ASMR (if stdweight provided)  ## -----------------------------  ## Age-specific projected COUNTS (deaths): list of length n_age  ## Each element: matrix with columns like 0.025Q/mean/0.975Q/sd (after qapc)  agespec_deaths <- agespec.proj(fit)  ## Example: projected deaths for age group 15–19  deaths_15_19 <- as.data.frame(agespec_deaths[[1]])  deaths_15_19$year <- years_all  ## Total deaths per year (simple sum of posterior means across age groups)  ## NOTE: summing quantiles across ages ignores between-age dependence; use with caution.  deaths_mean_mat <- sapply(agespec_deaths, function(m) m[, "mean"])  total_deaths_mean <- rowSums(deaths_mean_mat)  ## If you provided stdweight in BAPC(), you can extract age-standardized rates/projections:  ## asmr <- as.data.frame(agestd.rate(fit)) # age-standardized rates (historical+projected)  ## asmr_proj_counts <- as.data.frame(agestd.proj(fit)) # age-standardized projected counts (if needed)  proj_df <- data.frame(  year = years_all,  total_deaths_mean = total_deaths_mean  )  ## proj_df can be used to plot historical (1990–2021) and projected (2022–2040) totals. |
| --- |
